# Supplementary material for: Pentacyclic Triterpene Profile and Its Biosynthetic Pathway in Cecropia telenitida as a Prospective Dietary Supplement
Source: Molecules. 2021 Feb 18;26(4):1064. doi: 10.3390/molecules26041064 (PMC7922737; doi:10.3390/molecules26041064)
Supplement: Supplementary file 1 [file molecules-26-01064-s001.pdf]

Supplementary information: Pentacyclic triterpene profile and its biosynthetic pathway in *Cecropia telenitida* as a prospect type 2 diabetes dietary supplement

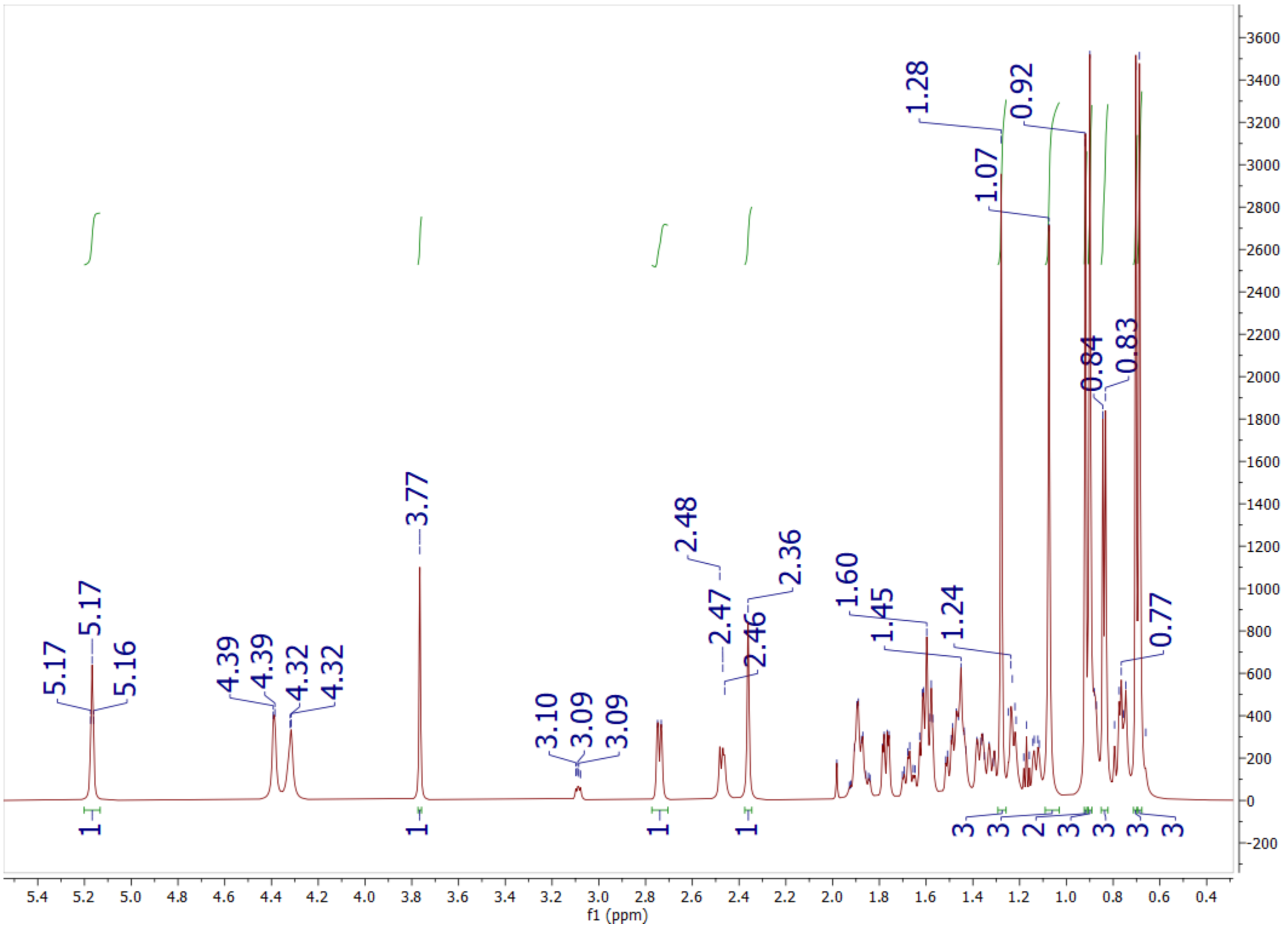

Figure S1. R_t 17.94 min peak ^{1}H-NMR spectrum. Isoyarumic acid.

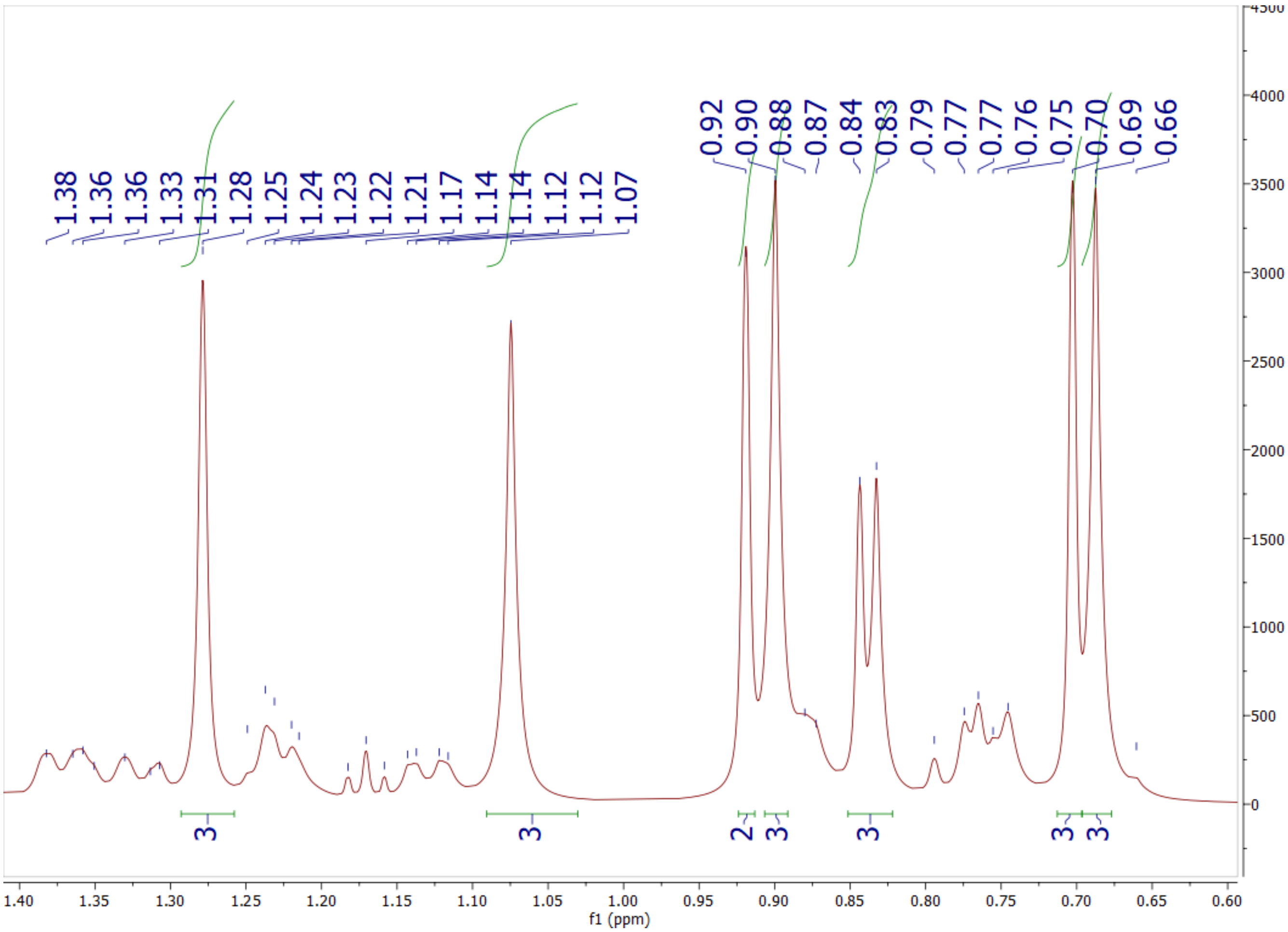

Figure S2. R_t 17.94 min peak ^{1}H-NMR spectrum, zoom over 0.60 to 1.40 ppm region. Isoyarumic acid

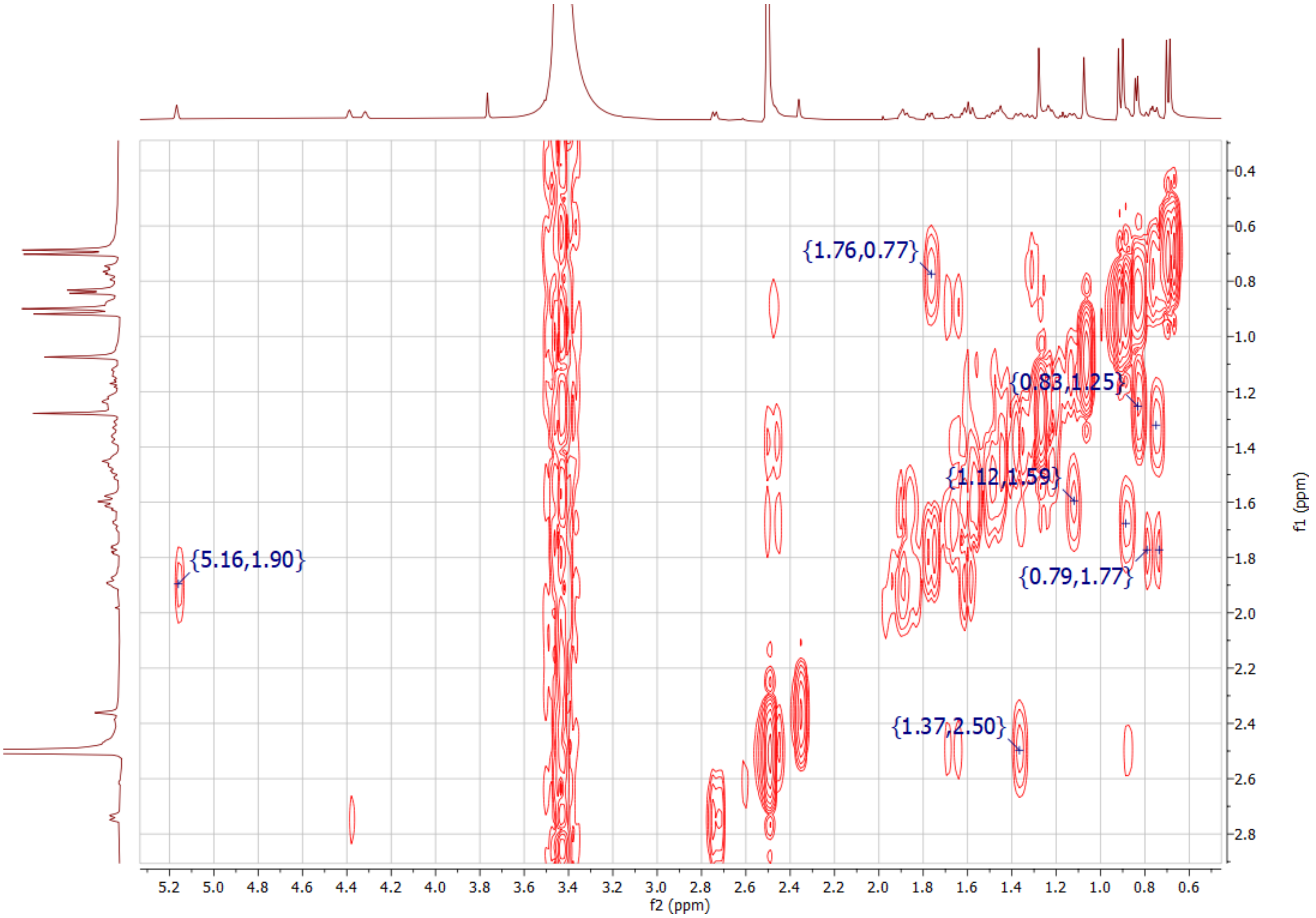

Figure S3. R_t 17.94 min peak ^1H-^1H NMR correlation (COSY) spectrum. Isoyarumic acid.

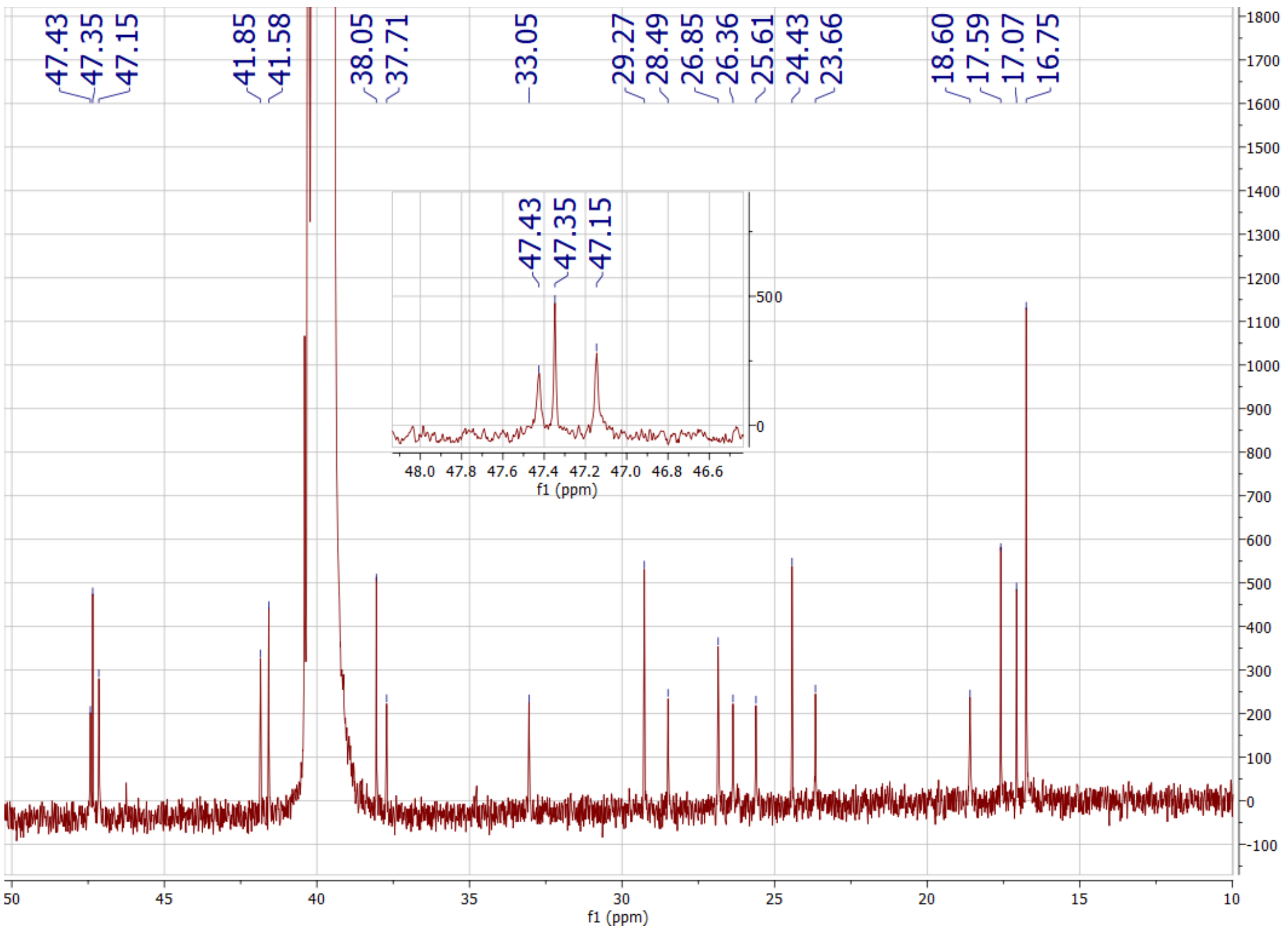

Figure S4. R_t 17.94 min peak ^{13}C-NMR spectrum, zoom over 10 to 50 ppm region. Isoyarumic acid.

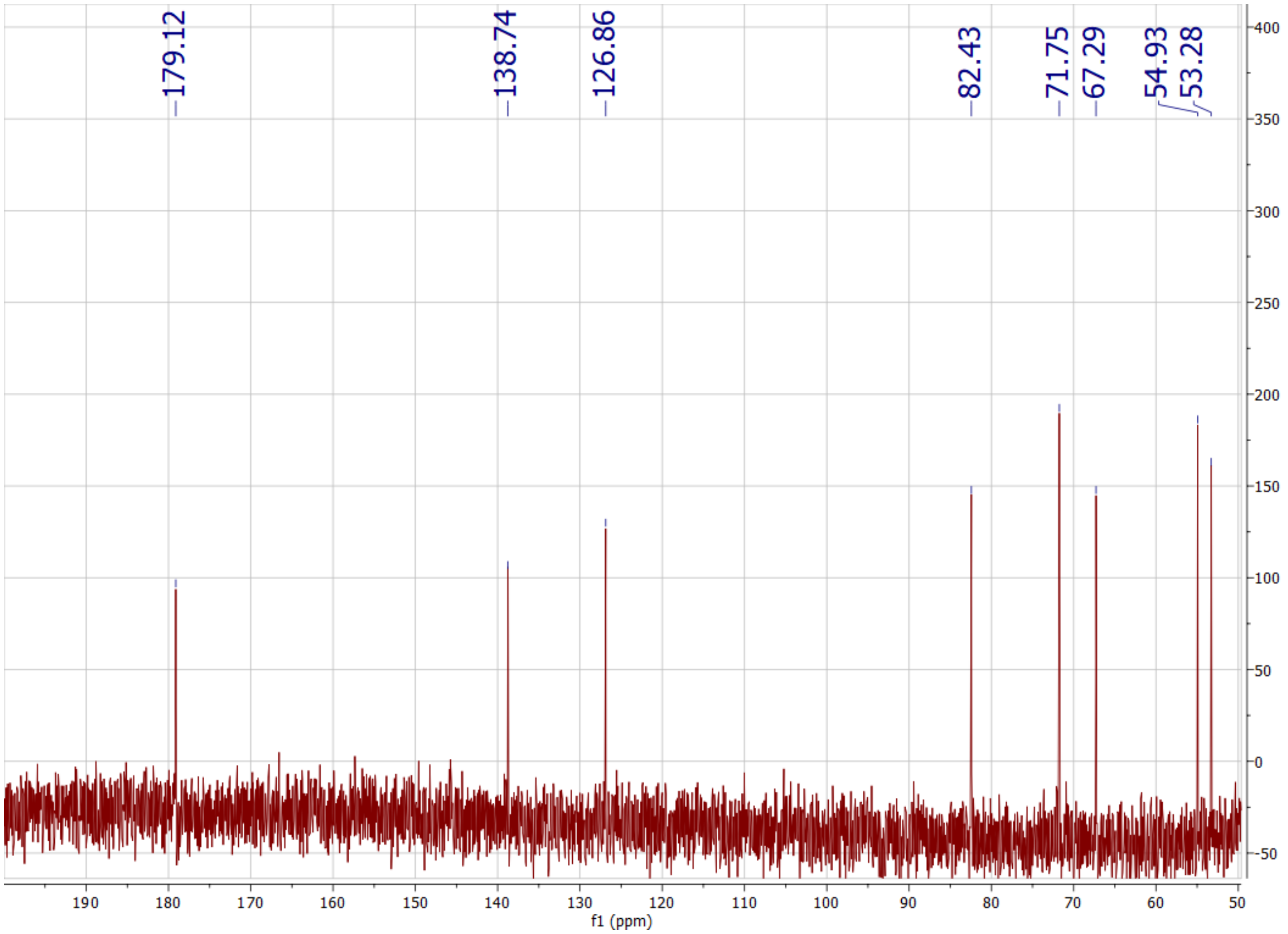

Figure S5. R_t 17.94 min peak ^{13}C-NMR spectrum, zoom over 50 to 200 ppm region. Isoyarumic acid.

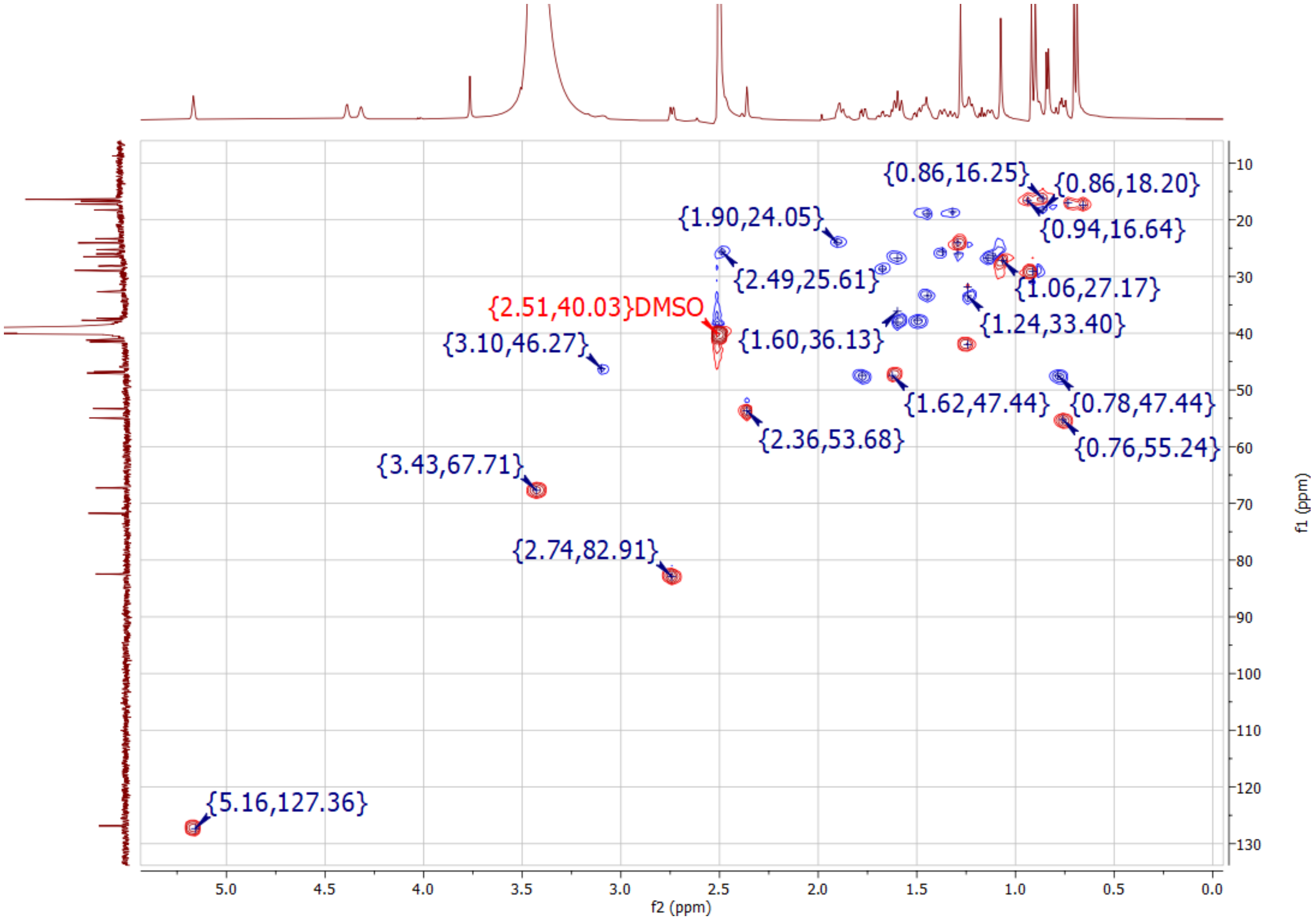

Figure S6. R_t 17.94 min peak ^{1}H-^{13}C NMR correlation (HSQC) spectrum. Isoyarumic acid.

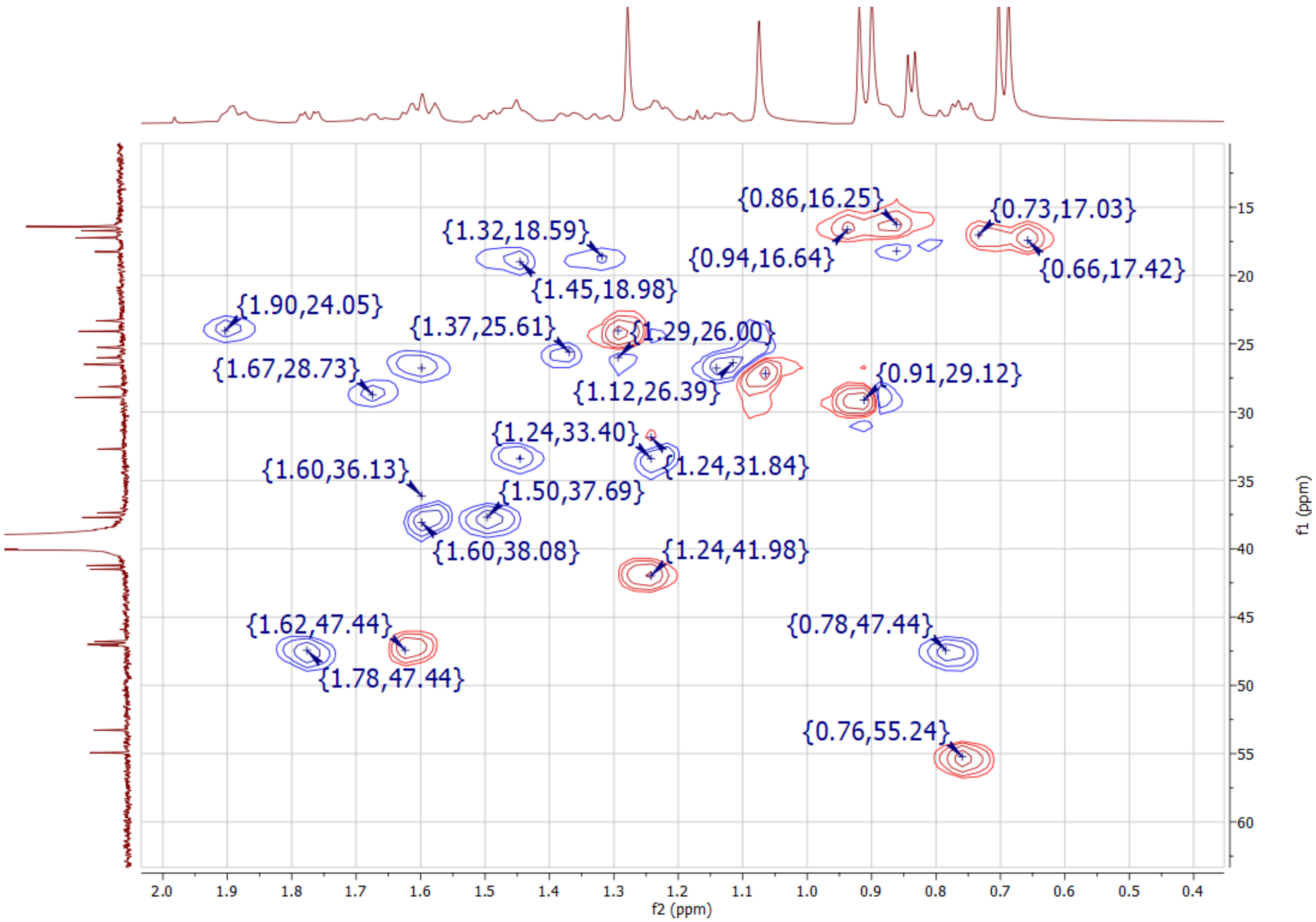

Figure S7. R_t 17.94 min peak ^{1}H-^{13}C NMR correlation (HSQC) spectrum, zoom over 15 to 60 ppm (f_1) and 0.4 to 2.0 ppm (f_2). Isoyarumic acid.

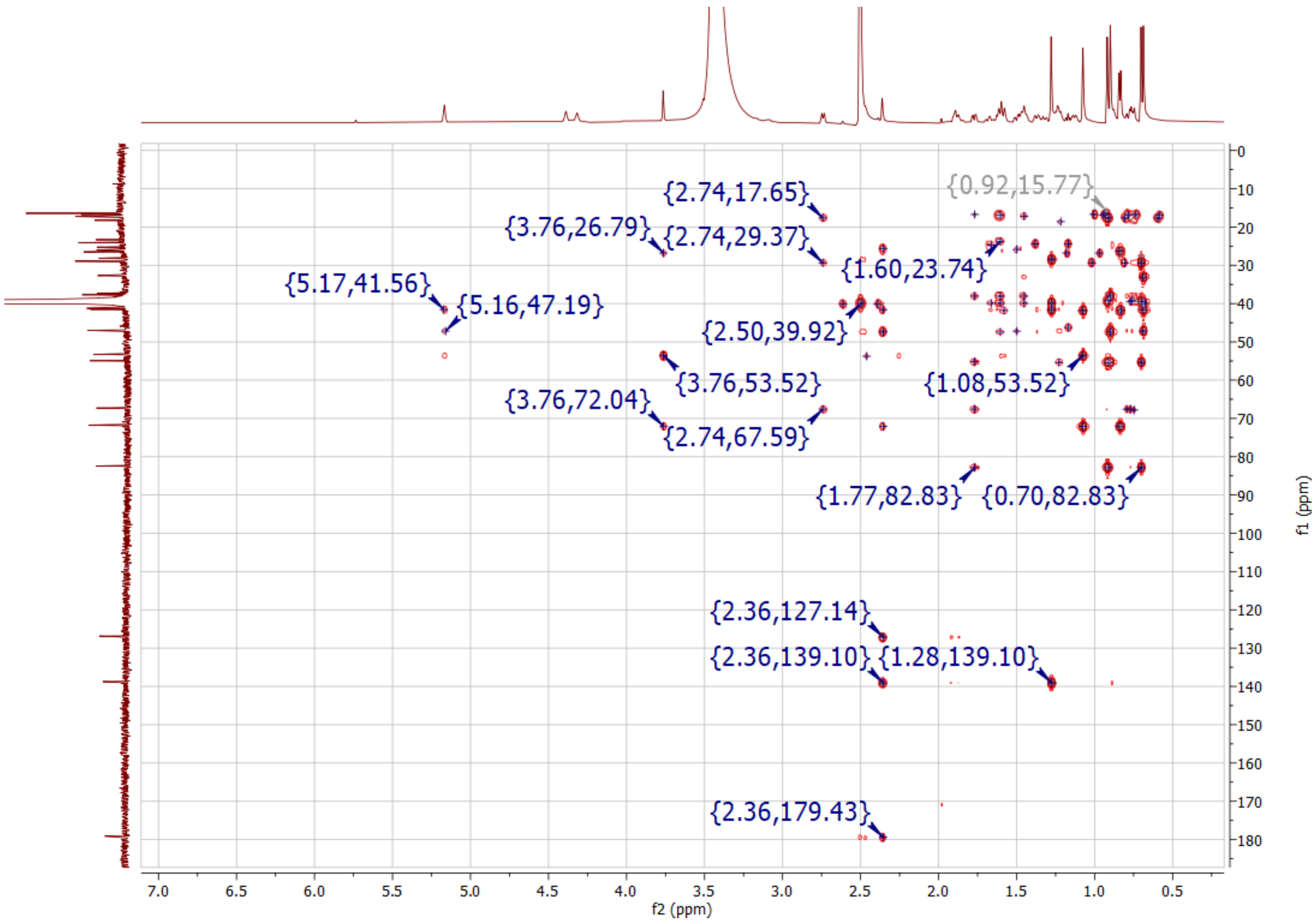

Figure S8. R_t 17.94 min peak ^{1}H-^{13}C NMR correlation (HMBC) spectrum. Isoyarumic acid.

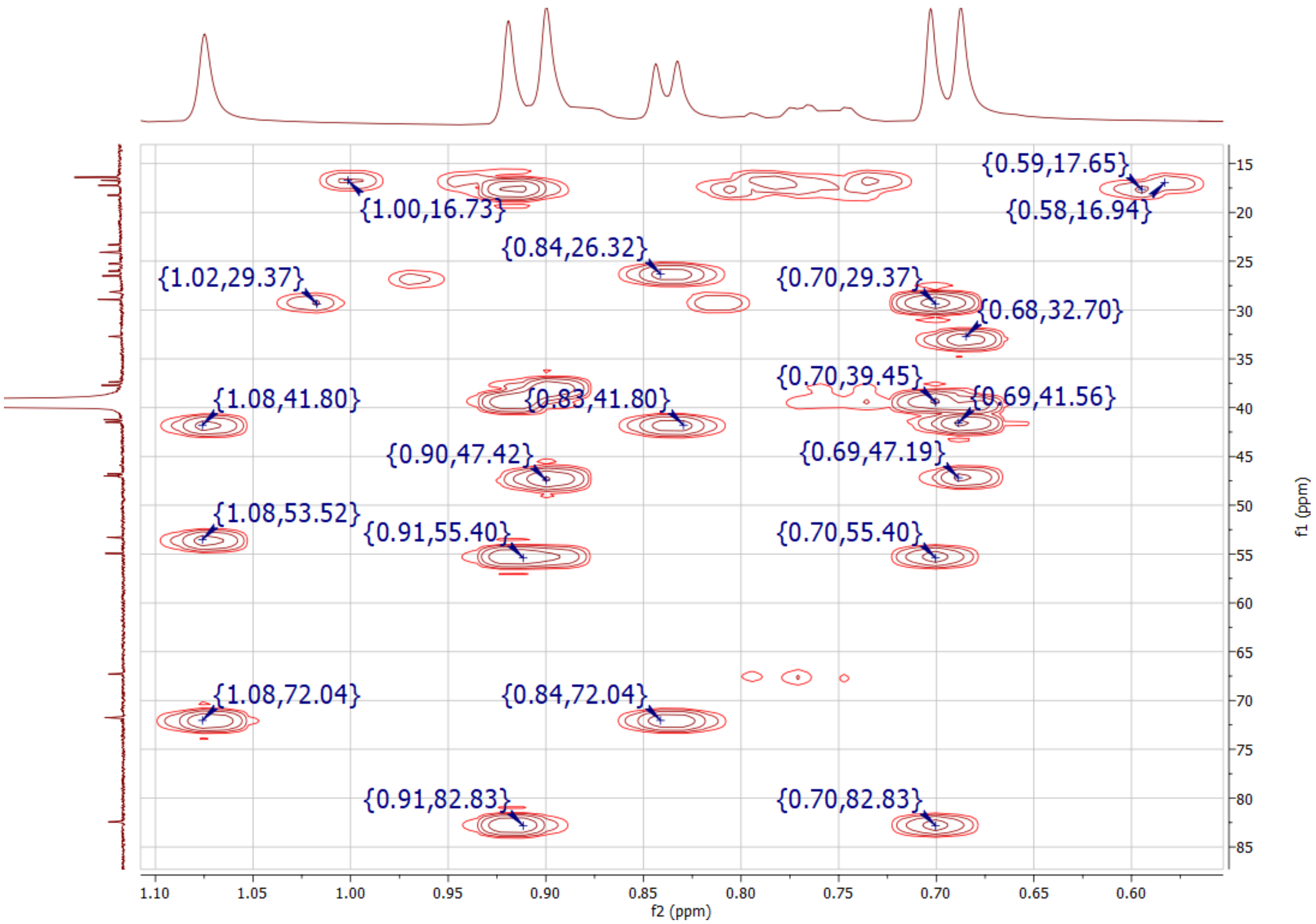

Figure S9. R_t 17.94 min peak ^{1}H-^{13}C NMR correlation (HMBC) spectrum, zoom over 15 to 85 ppm (f_1) and 0.5 to 1.10 ppm (f_2). Isoyarumic acid.

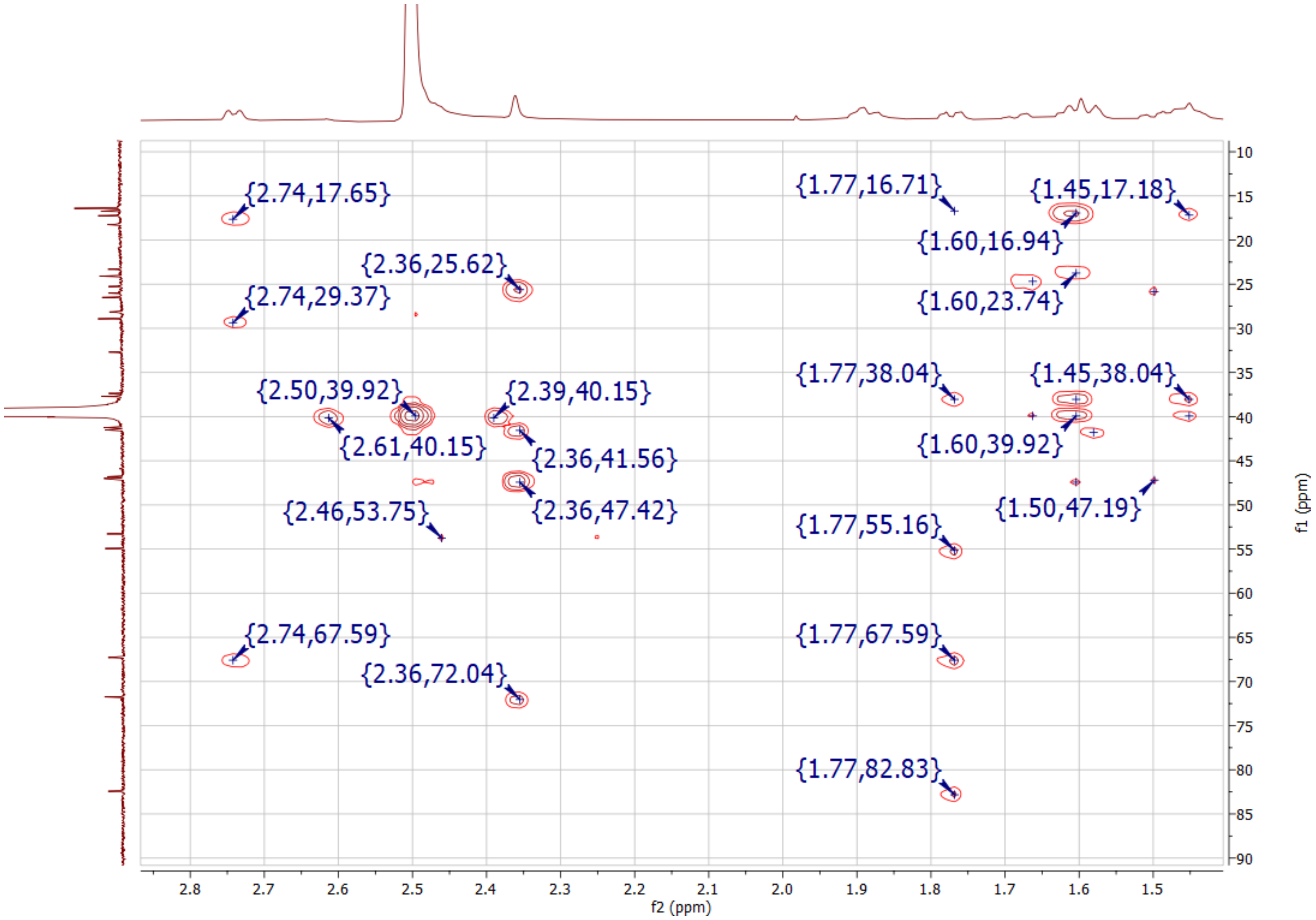

Figure S9. R_t 17.94 min peak ^{1}H-^{13}C NMR correlation (HMBC) spectrum, zoom over 10 to 90 ppm (f_1) and 1.0 to 2.8 ppm (f_2). Isoyarumic acid.

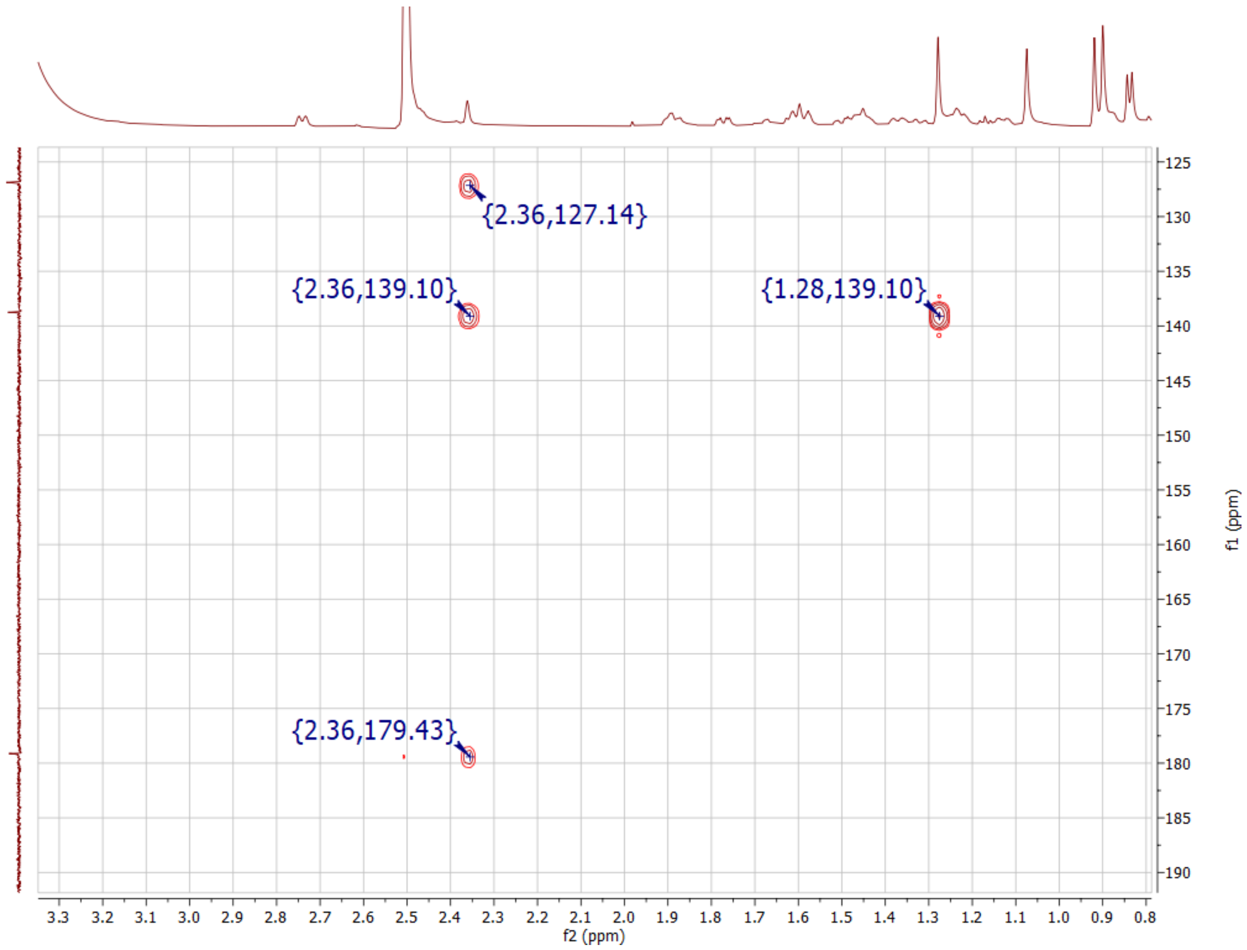

Figure S9. R_t 17.94 min peak ^{1}H-^{13}C NMR correlation (HMBC) spectrum, zoom over 125 to 190 ppm (f_1) and 0.8 to 3.3 ppm (f_2). Isoyarumic acid.

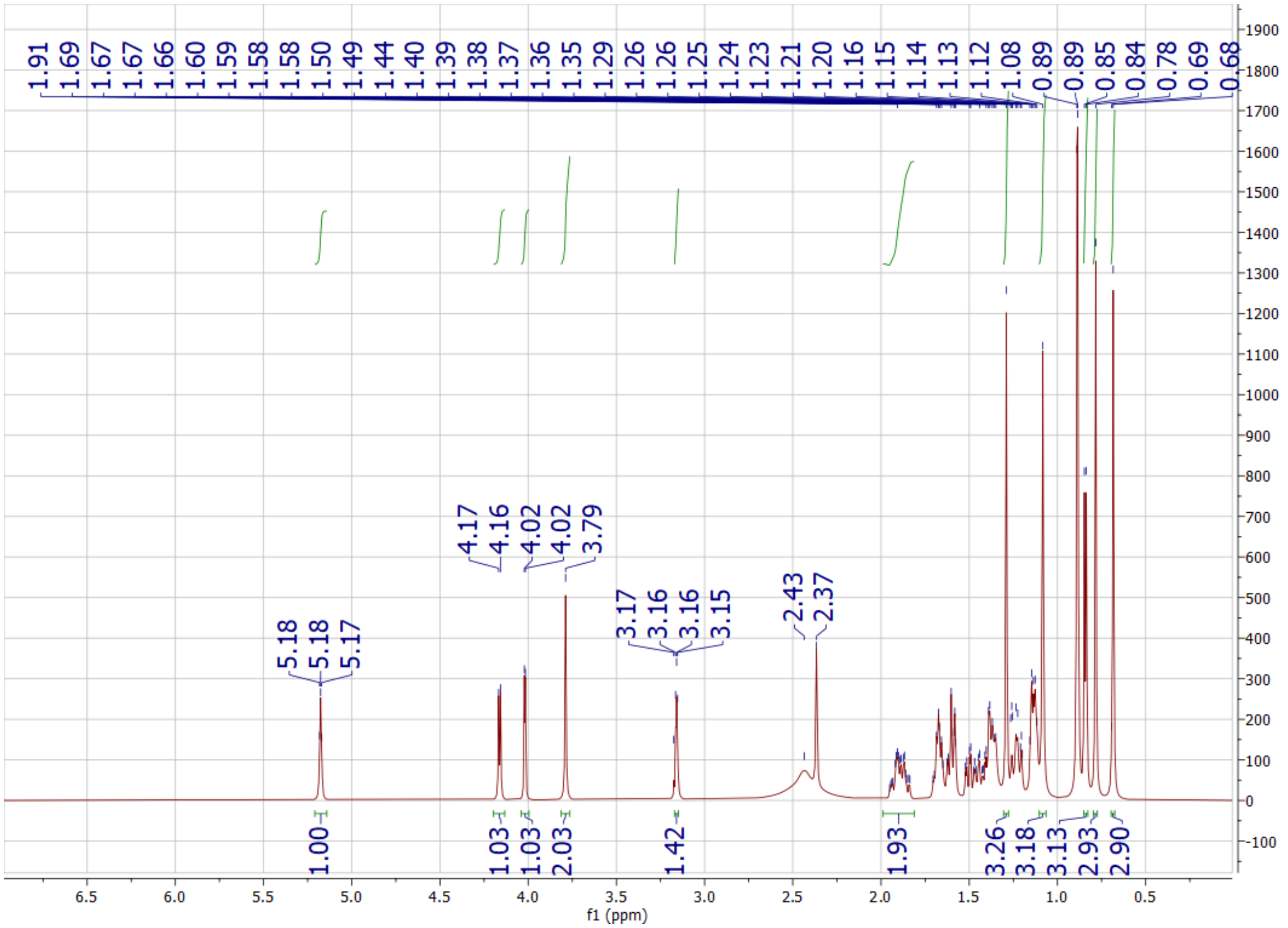

Figure S10. R_t 23.31 min peak ^{1}H-NMR spectrum. Tormentic acid.

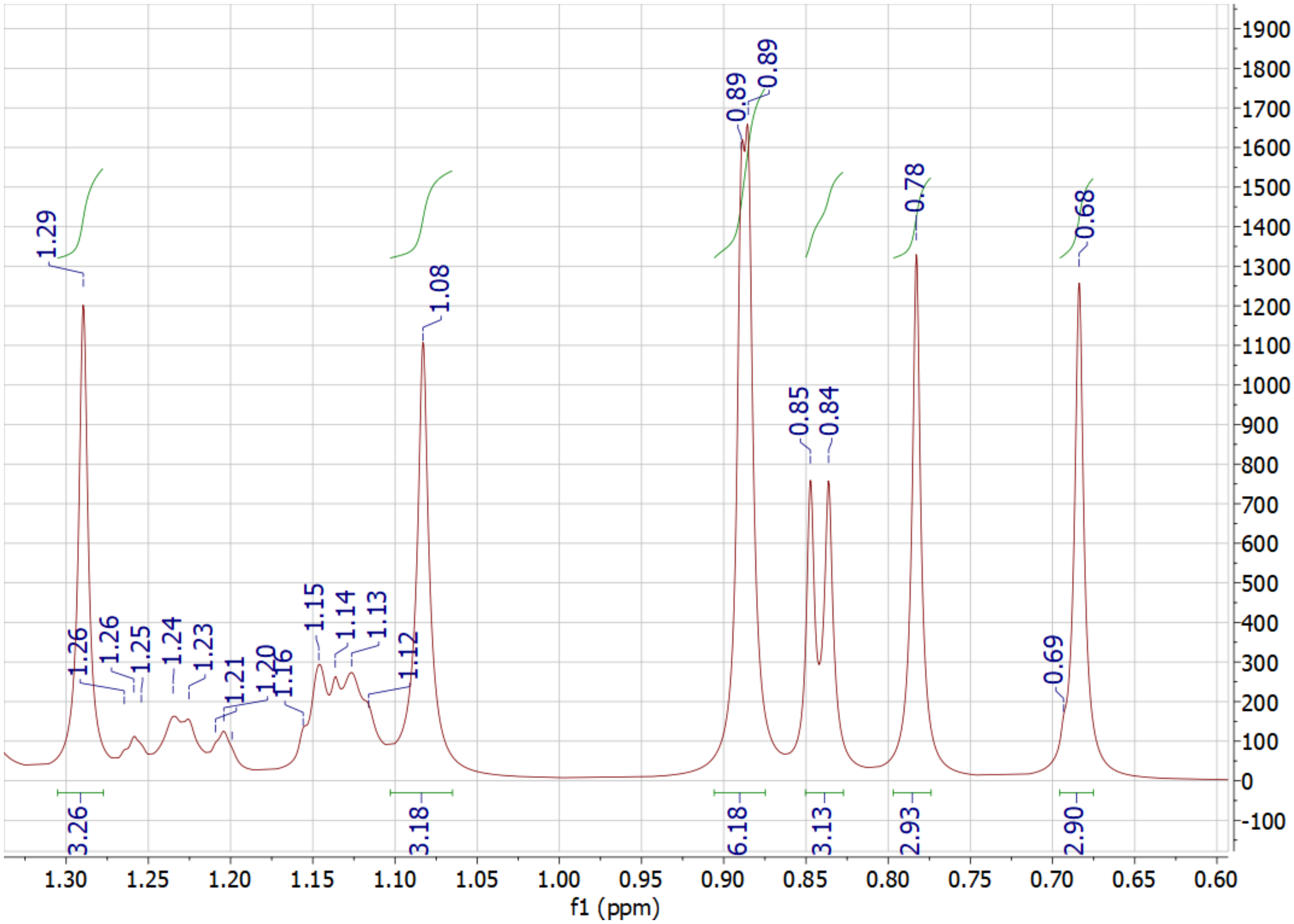

Figure S11. R_t 23.31 min peak ^{1}H-NMR spectrum, zoom over 0.60 to 1.40 ppm region. Tormentic acid.

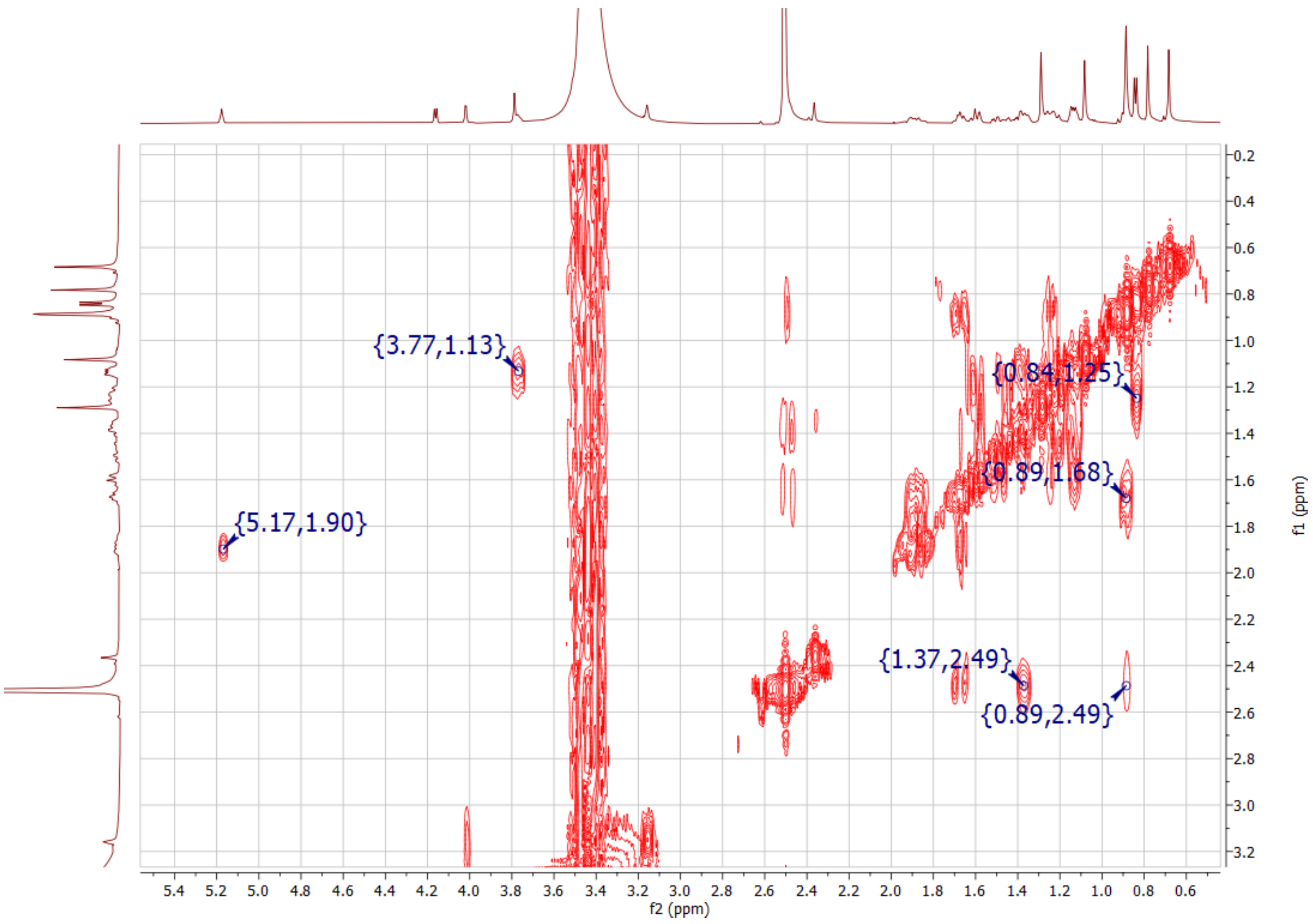

Figure S12. R_t 23.31 min peak ^{1}H-^{1}H NMR correlation (COSY) spectrum. Tormentic acid.

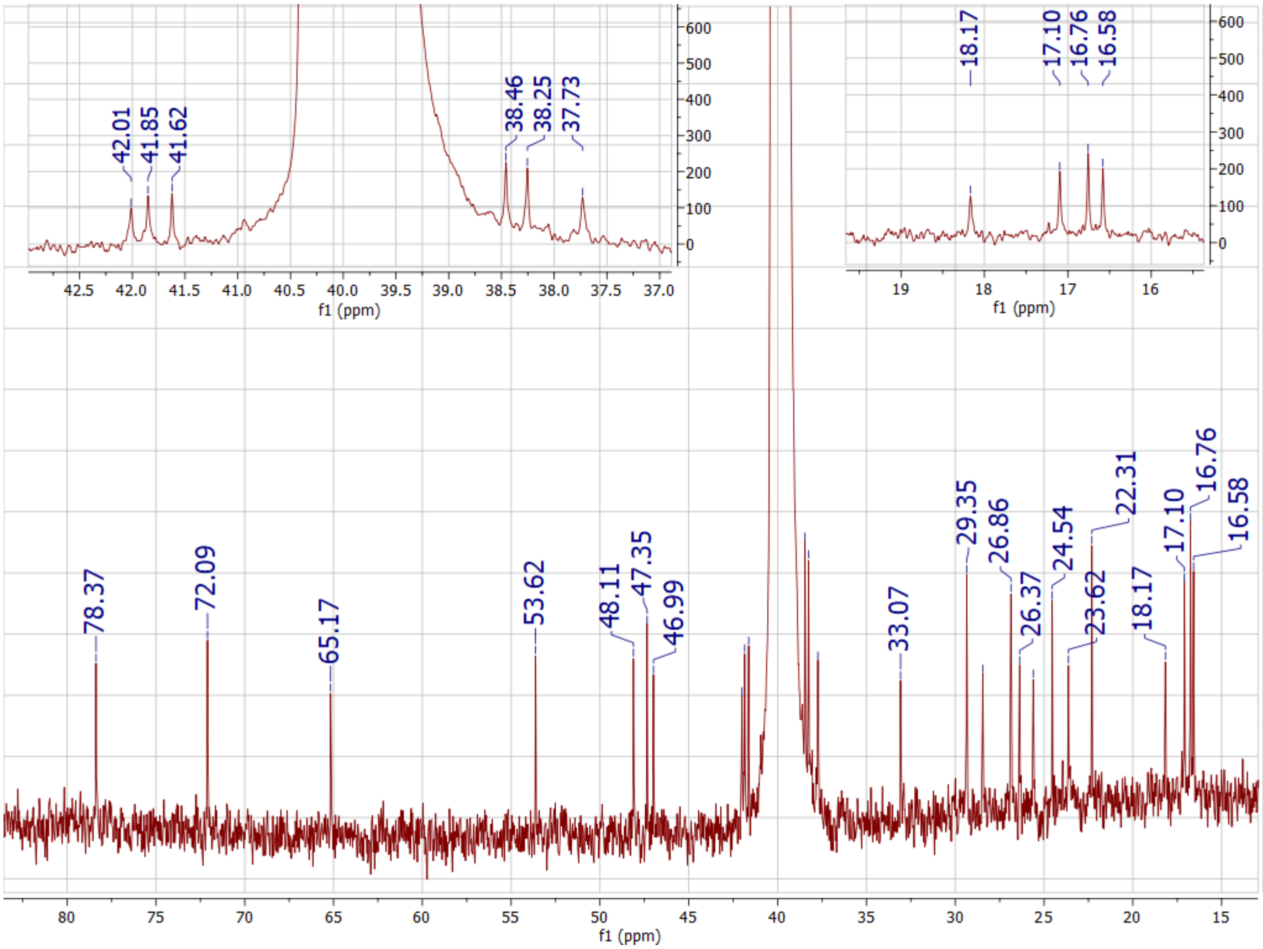

Figure S13. R_t 23.31 min peak ^{13}C-NMR spectrum, zoom over 15 to 80 ppm region. Tormentic acid.

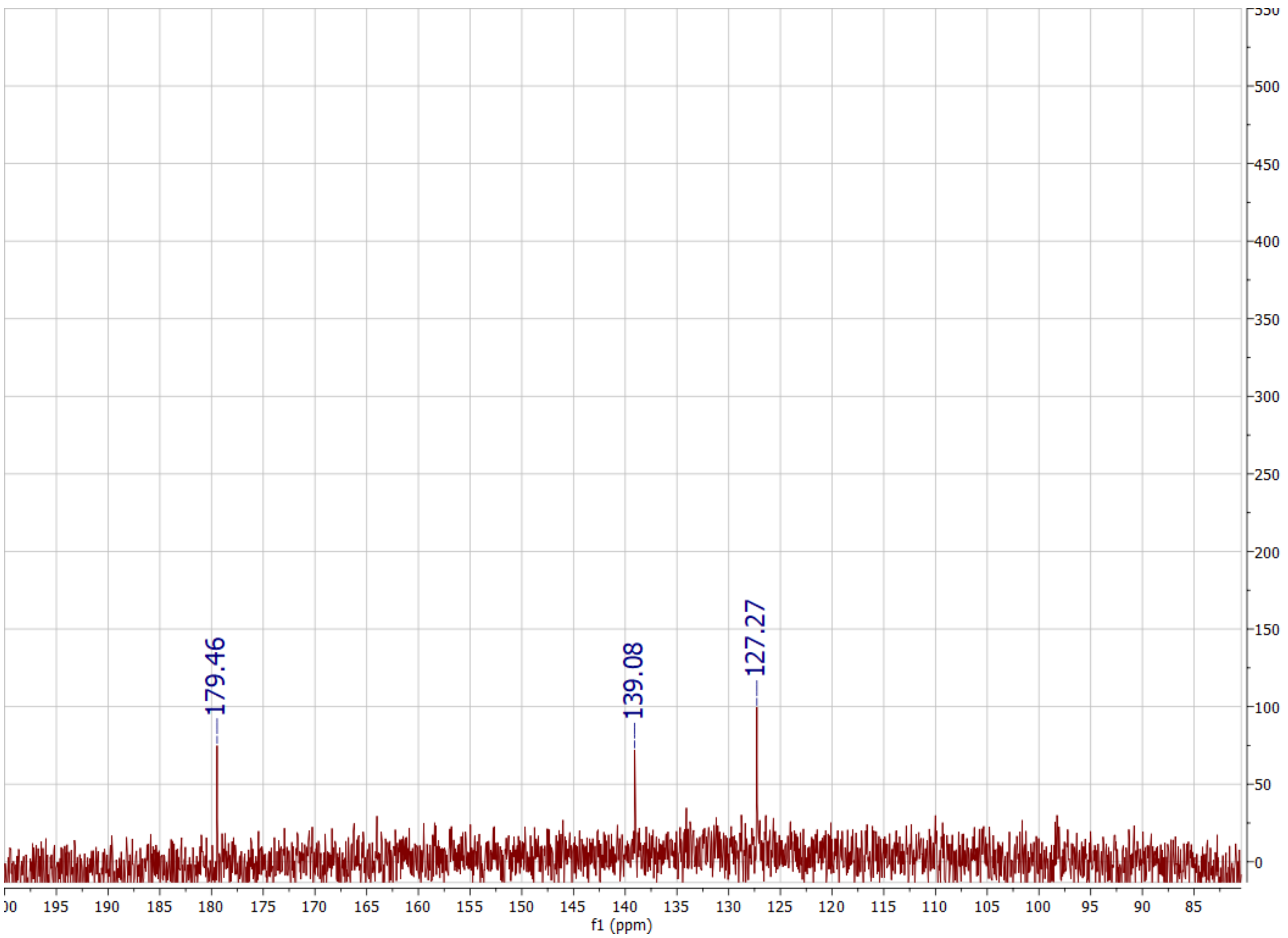

Figure S14. R_t 23.31 min peak ^{13}C-NMR spectrum, zoom over 80 to 200 ppm region. Tormentic acid.

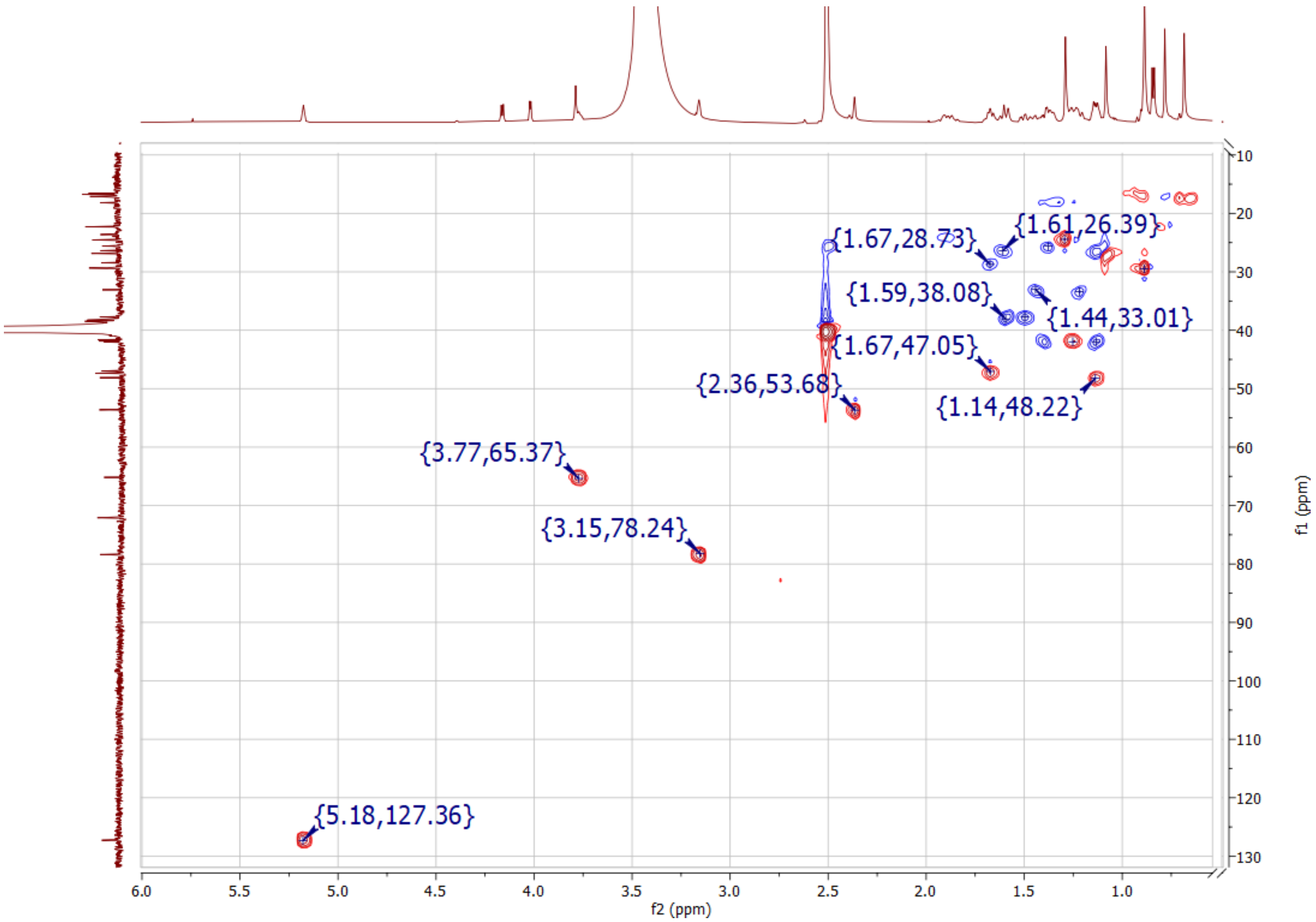

Figure S15. R_t 23.31 min peak ^{1}H-^{13}C NMR correlation (HSQC) spectrum. Tormentic acid.

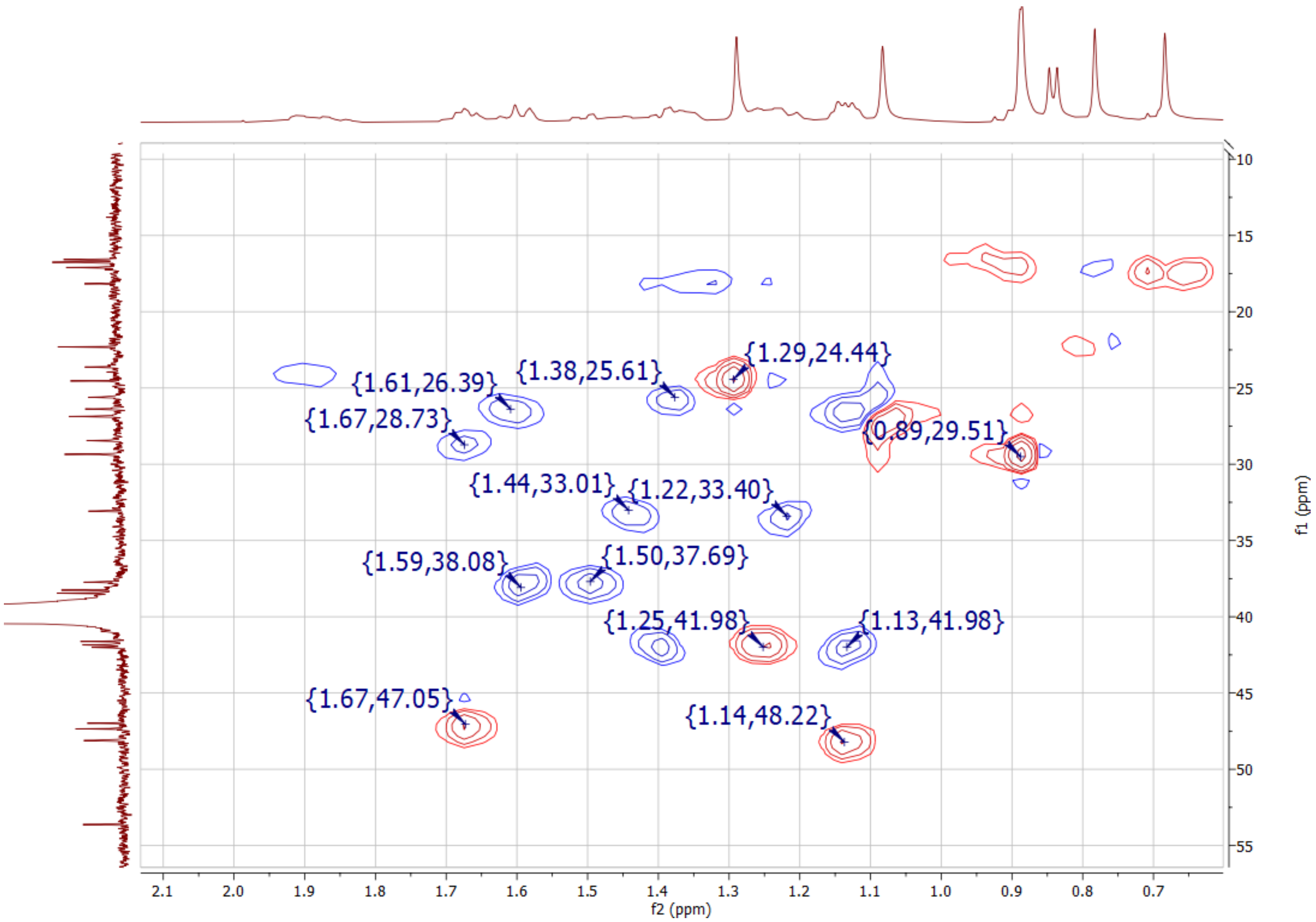

Figure S16. R_t 23.31 min peak ^{1}H-^{13}C NMR correlation (HSQC) spectrum, zoom over 10 to 55 ppm (f_1) and 0.6 to 2.1 ppm (f_2). Tormentic acid.

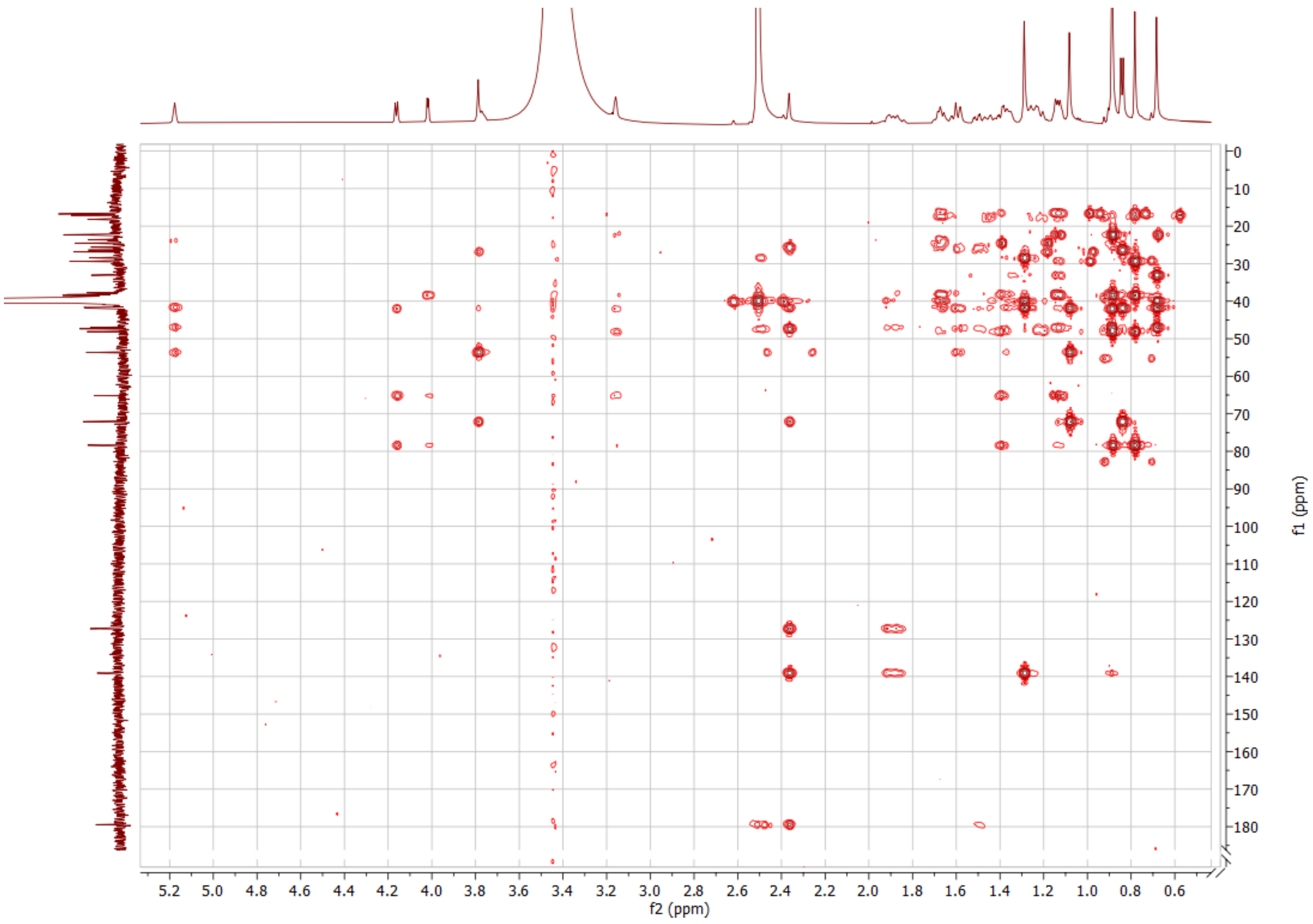

Figure S17. R_t 23.31 min peak ^{1}H-^{13}C NMR correlation (HMBC) spectrum. Tormentic acid.

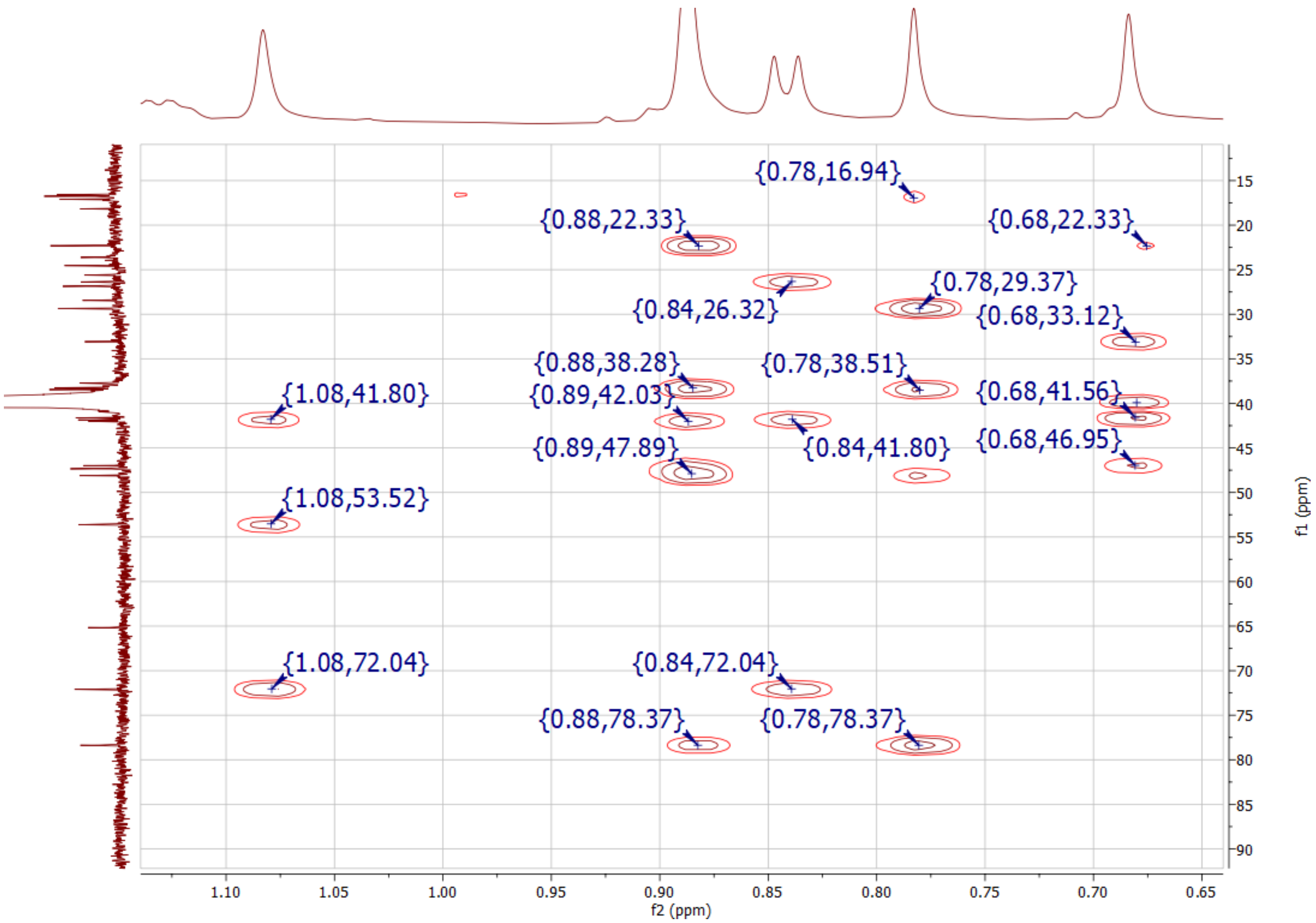

Figure S18. R_t 23.31 min peak ^{1}H-^{13}C NMR correlation (HMBC) spectrum, zoom over 15 to 90 ppm (f_1) and 0.65 to 1.10 ppm (f_2). Tormentic acid.

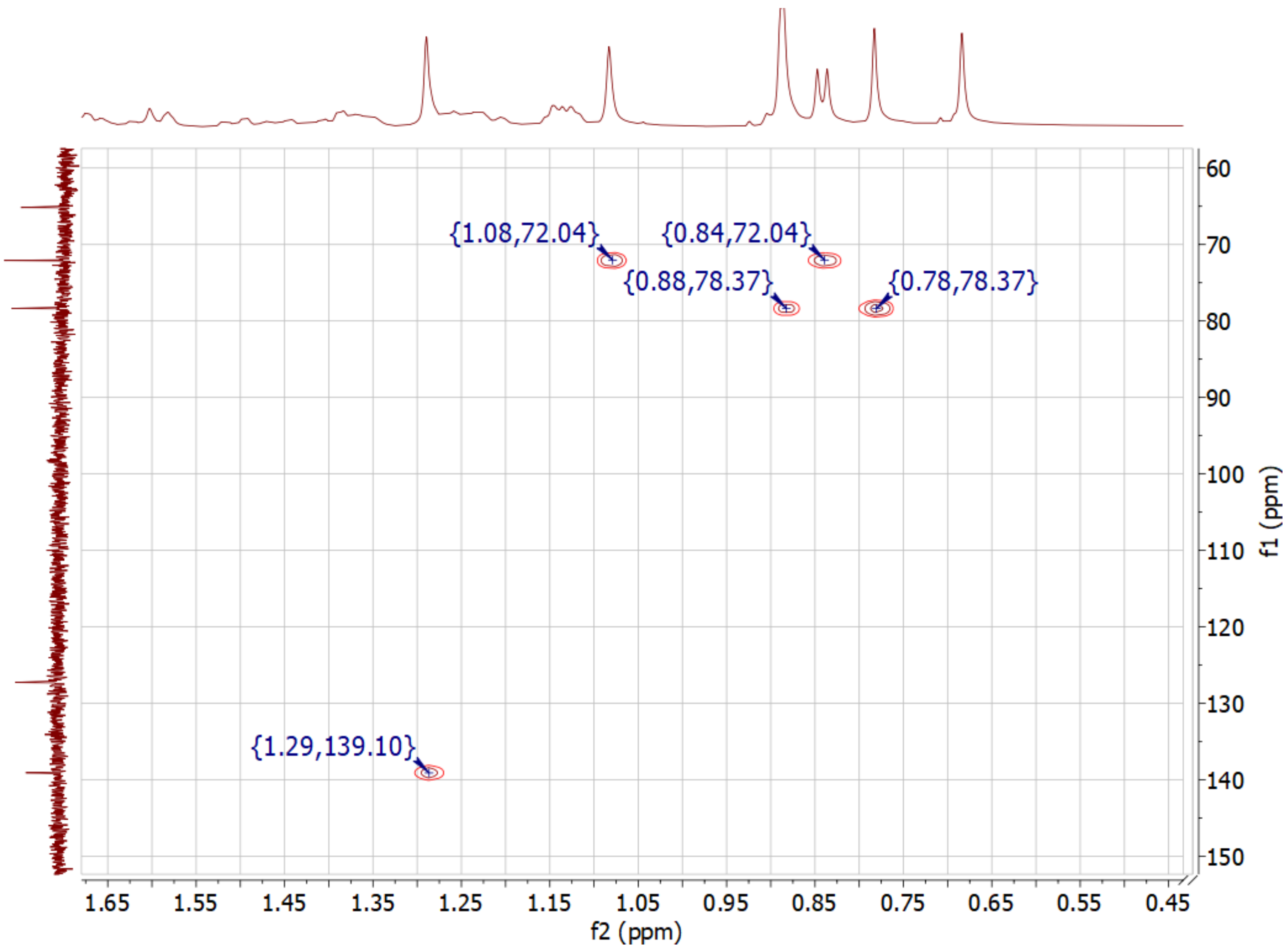

Figure S19. R_t 23.31 min peak ^{1}H-^{13}C NMR correlation (HMBC) spectrum, zoom over 60 to 150 ppm (f_1) and 0.45 to 1.65 ppm (f_2). Tormentic acid.

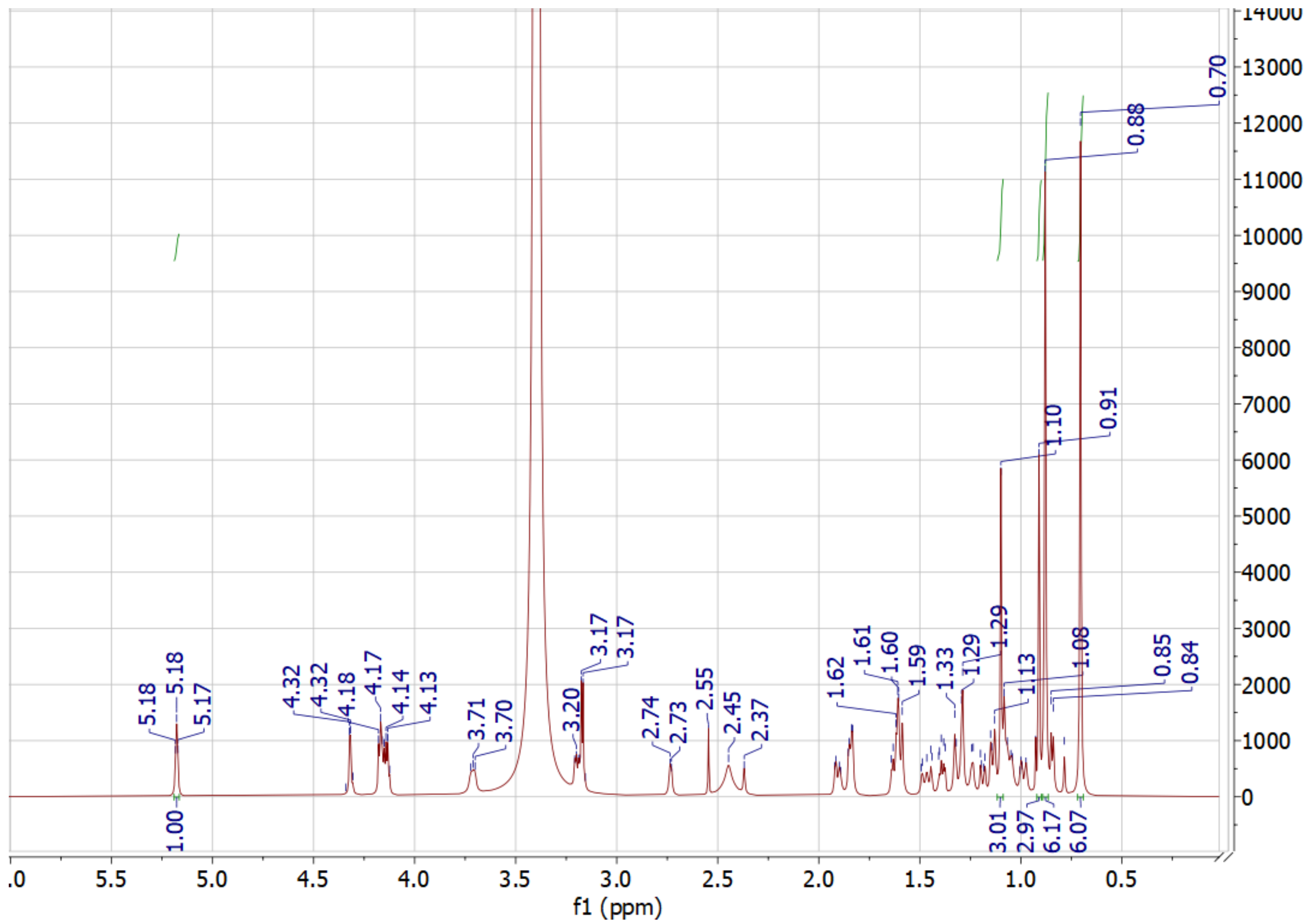

Figure S20. R_t 27.47 min peak ^{1}H-NMR spectrum. Hederagenic acid.

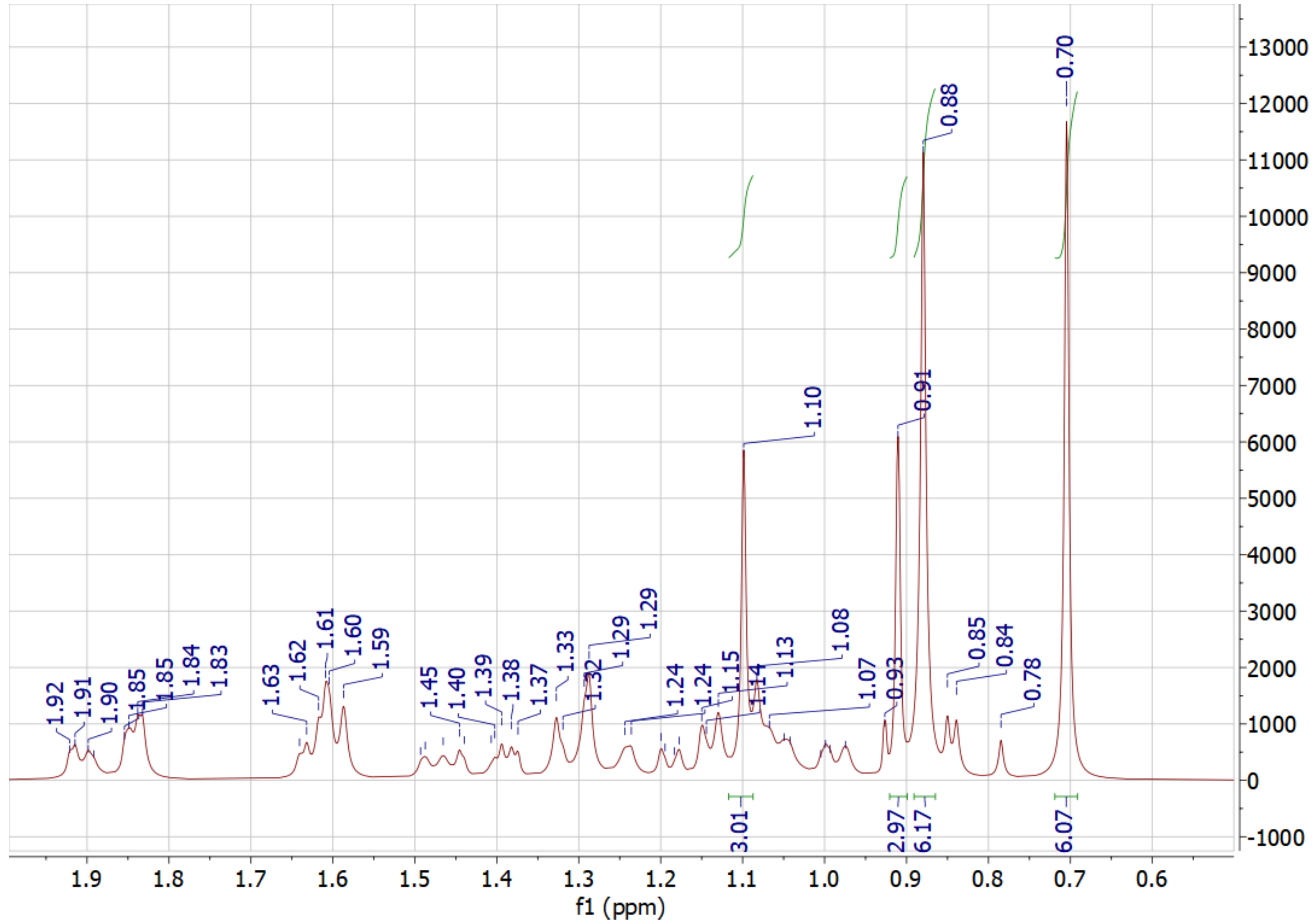

Figure S21. R_t 27.47 min peak ^{1}H-NMR spectrum, zoom from 0.5 to 2.0 ppm. Hederagenic acid.

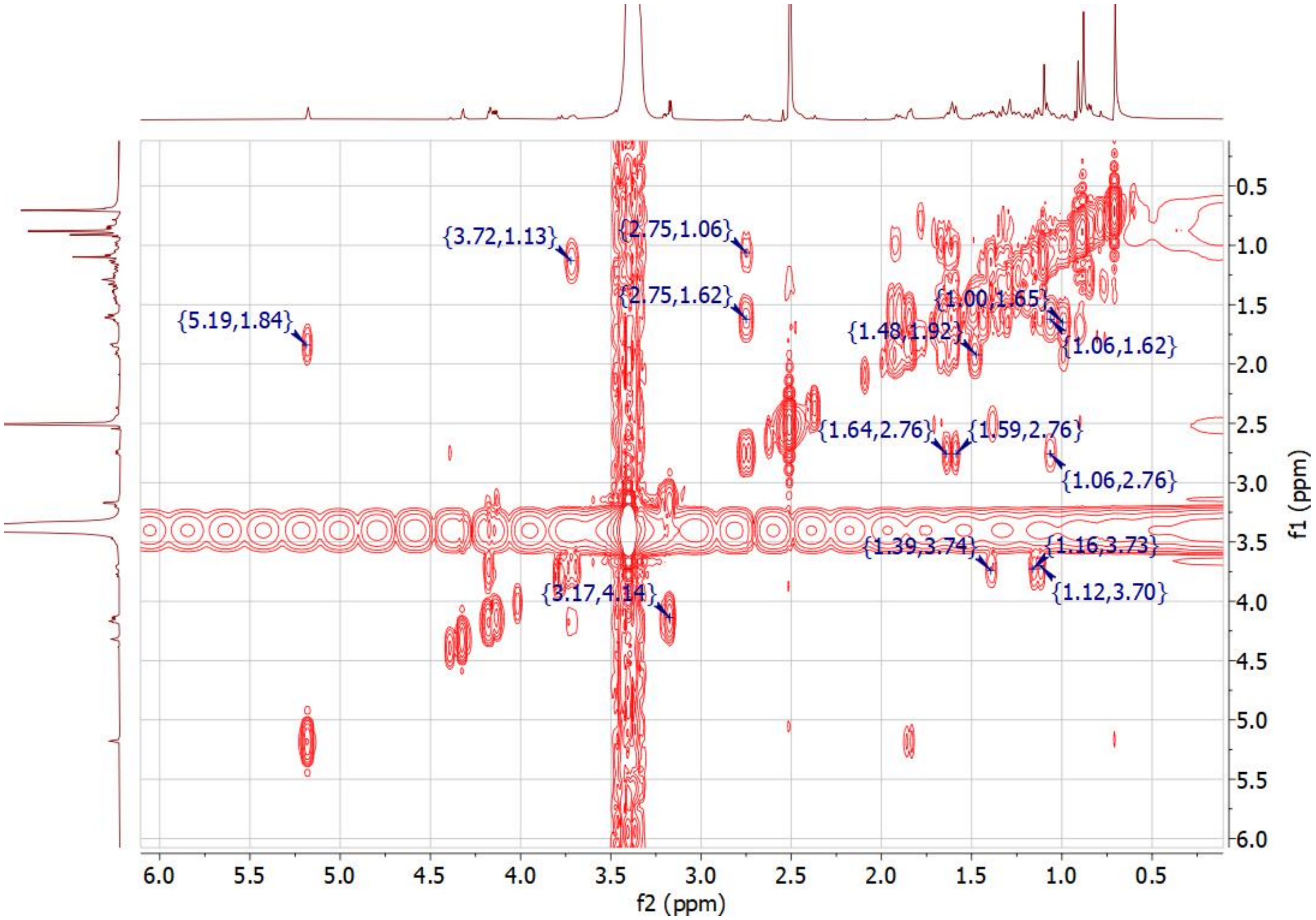

Figure S22. R_t 27.47 min peak ^{1}H-^{1}H NMR correlation (COSY) spectrum. Hederagenic acid.

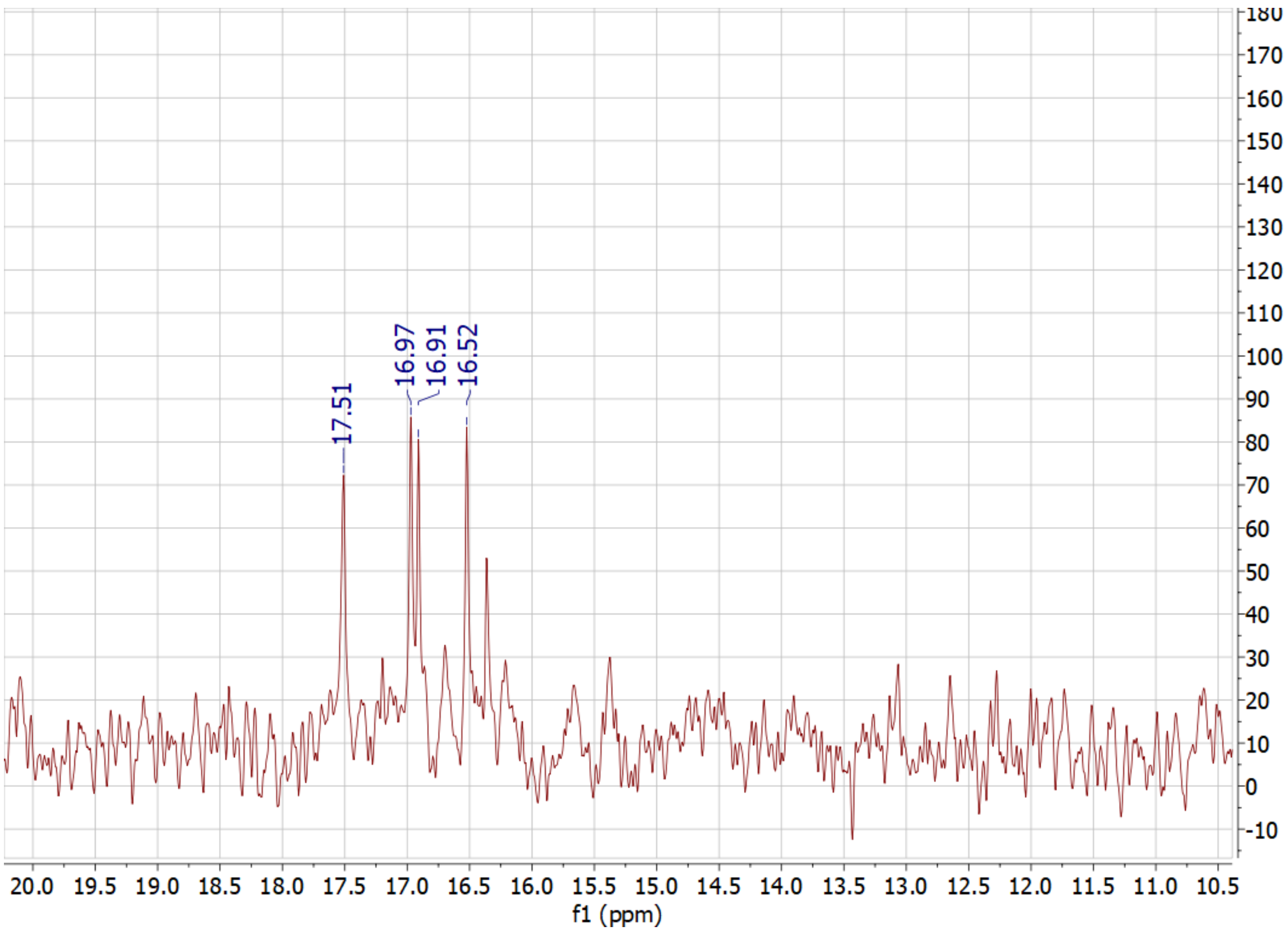

Figure S23. R_t 27.47 min peak ^{13}C-NMR spectrum, zoom over 10 to 20 ppm region. Hederagenic acid.

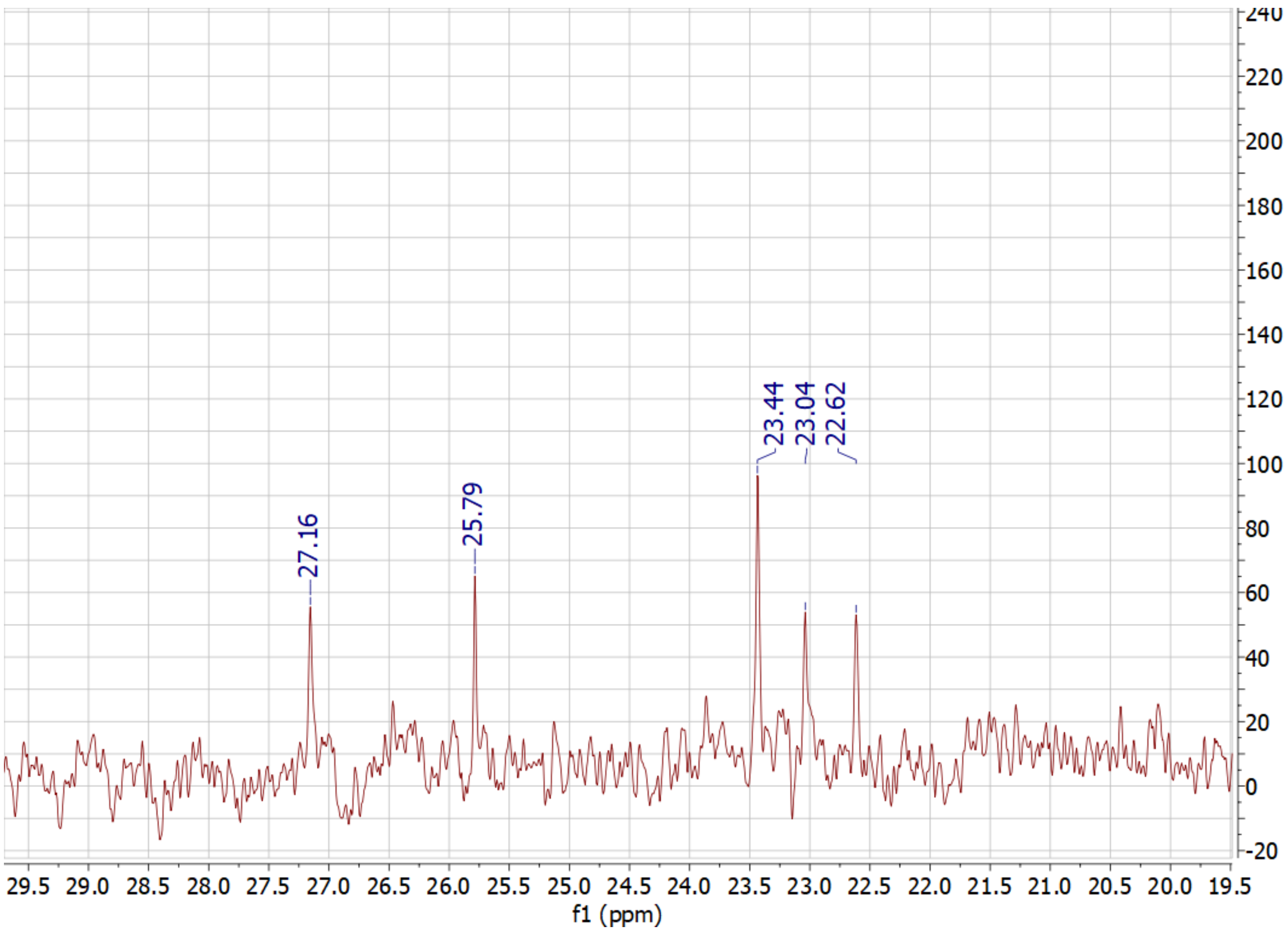

Figure S24. R_t 27.47 min peak ^{13}C-NMR spectrum, zoom over 20 to 30 ppm region. Hederagenic acid.

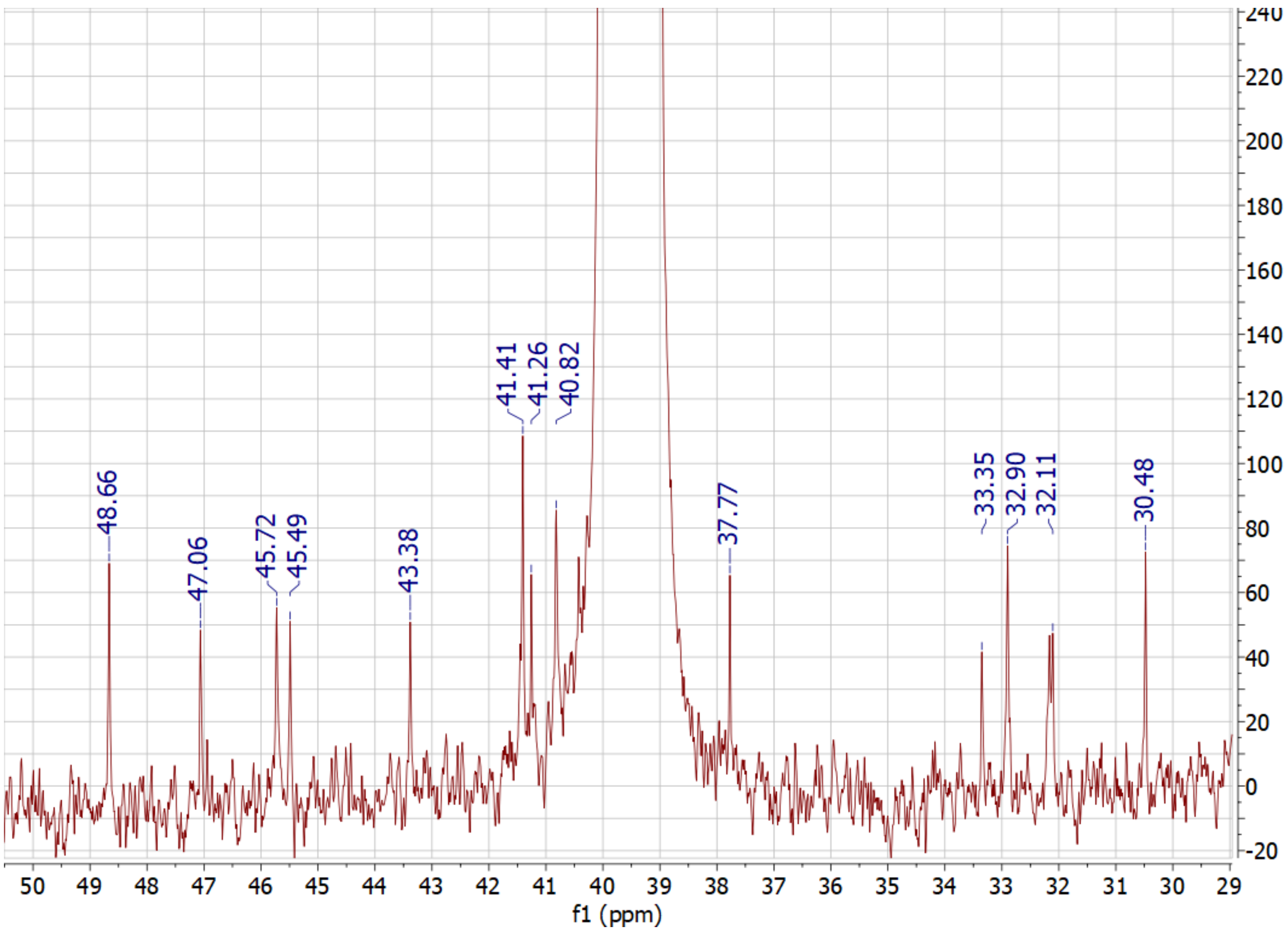

Figure S25. R_t 27.47 min peak ^{13}C-NMR spectrum, zoom over 30 to 50 ppm region. Hederagenic acid.

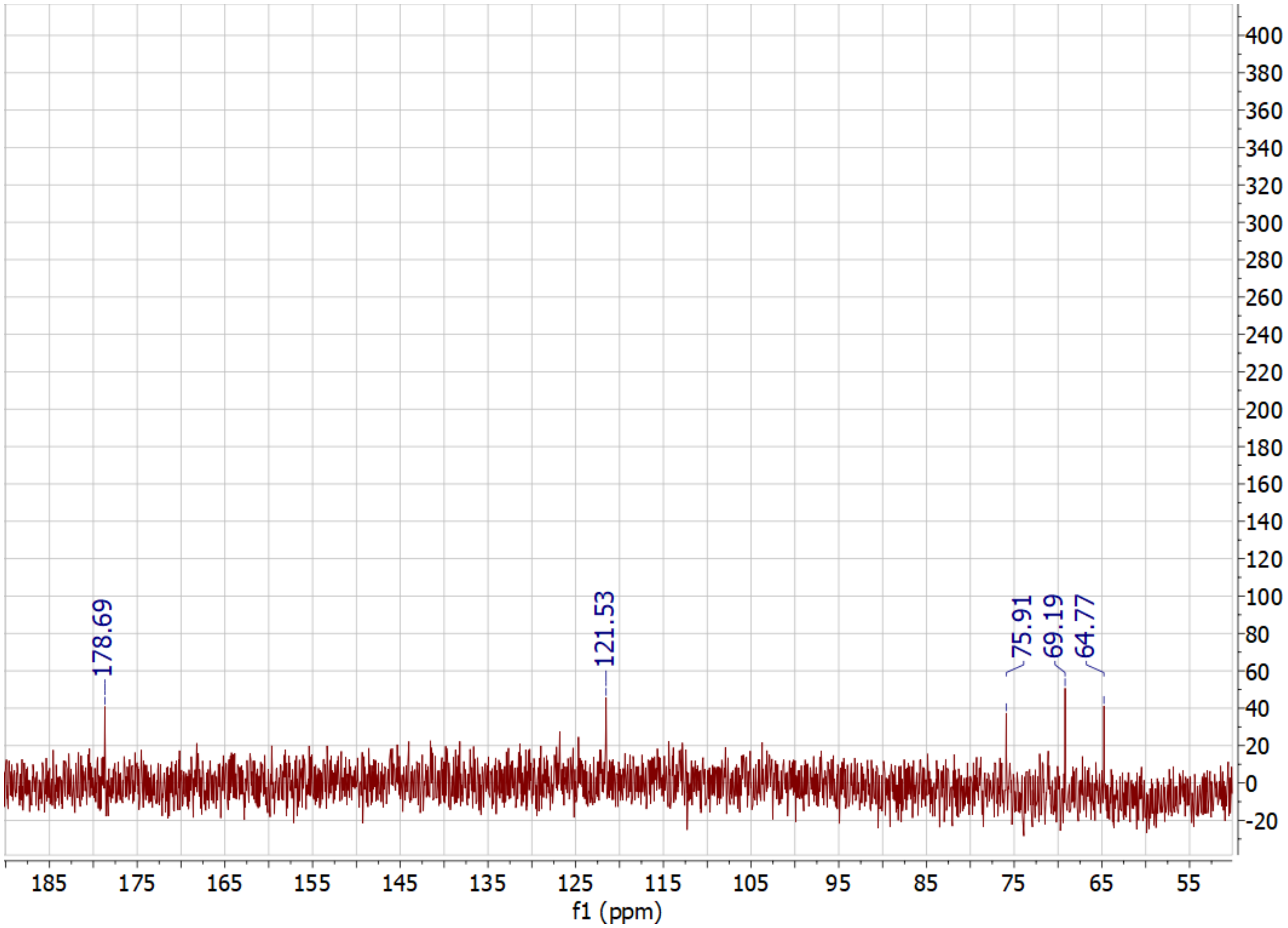

Figure S26. R_t 27.47 min peak ^{13}C-NMR spectrum, zoom over 50 to 185 ppm region. Hederagenic acid.

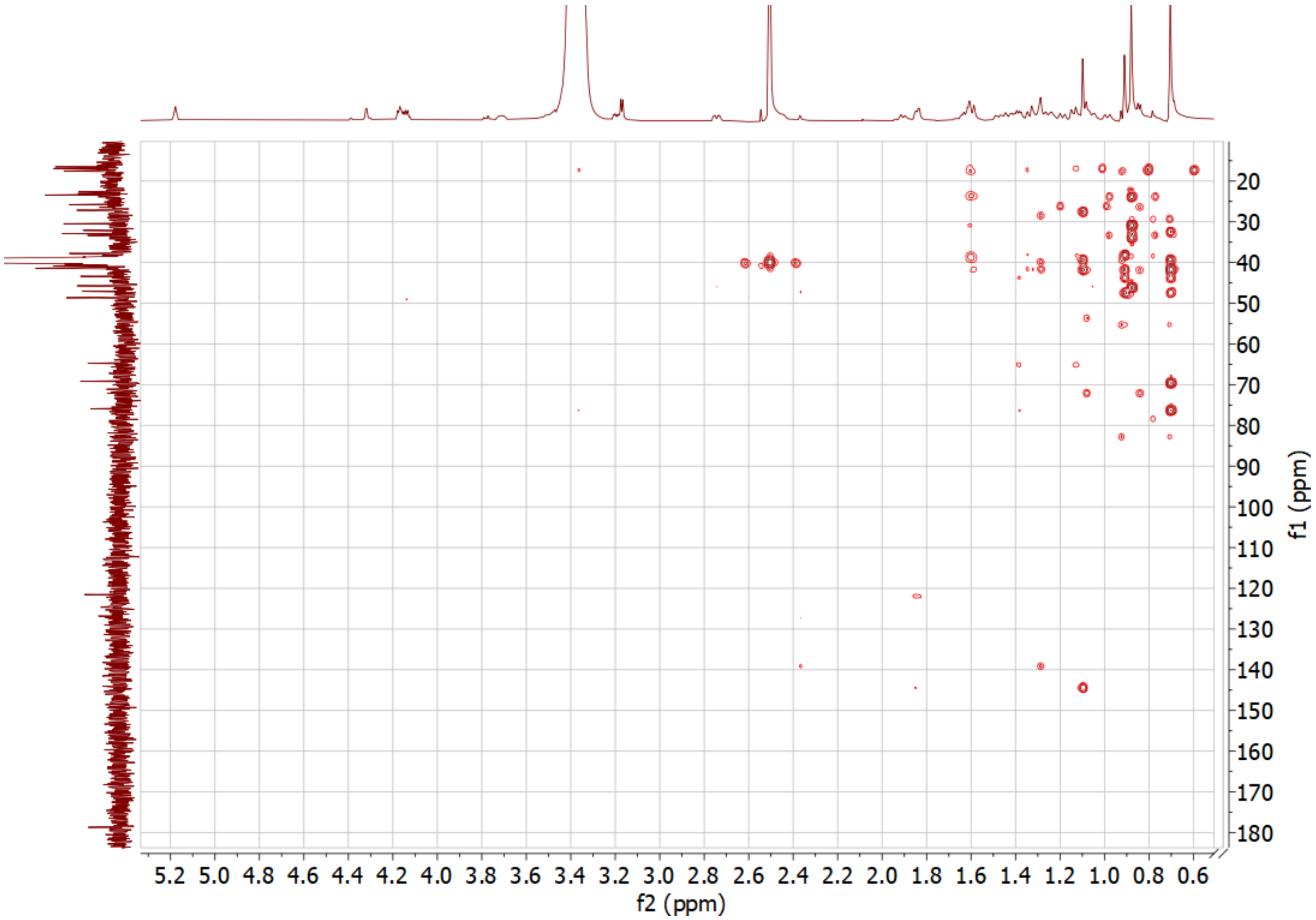

Figure S27. R_t 27.47 min peak ^{1}H-^{13}C NMR correlation (HMBC) spectrum. Hederagenic acid.

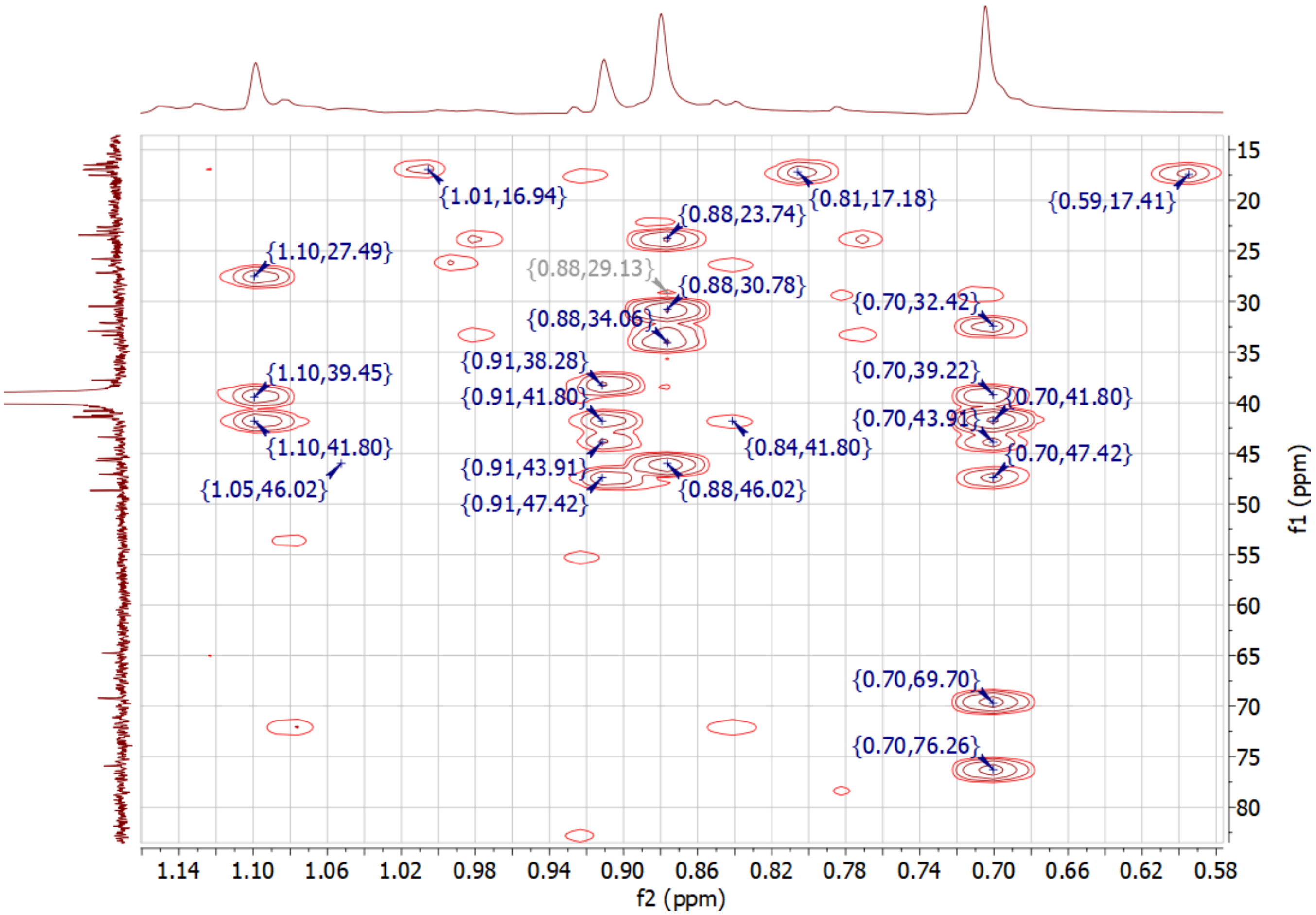

Figure S28. R_t 27.47 min peak ^{1}H-^{13}C NMR correlation (HMBC) spectrum, , zoom over 15 to 80 ppm (f_1) and 0.58 to 1.14 ppm (f_2). Hederagenic acid.

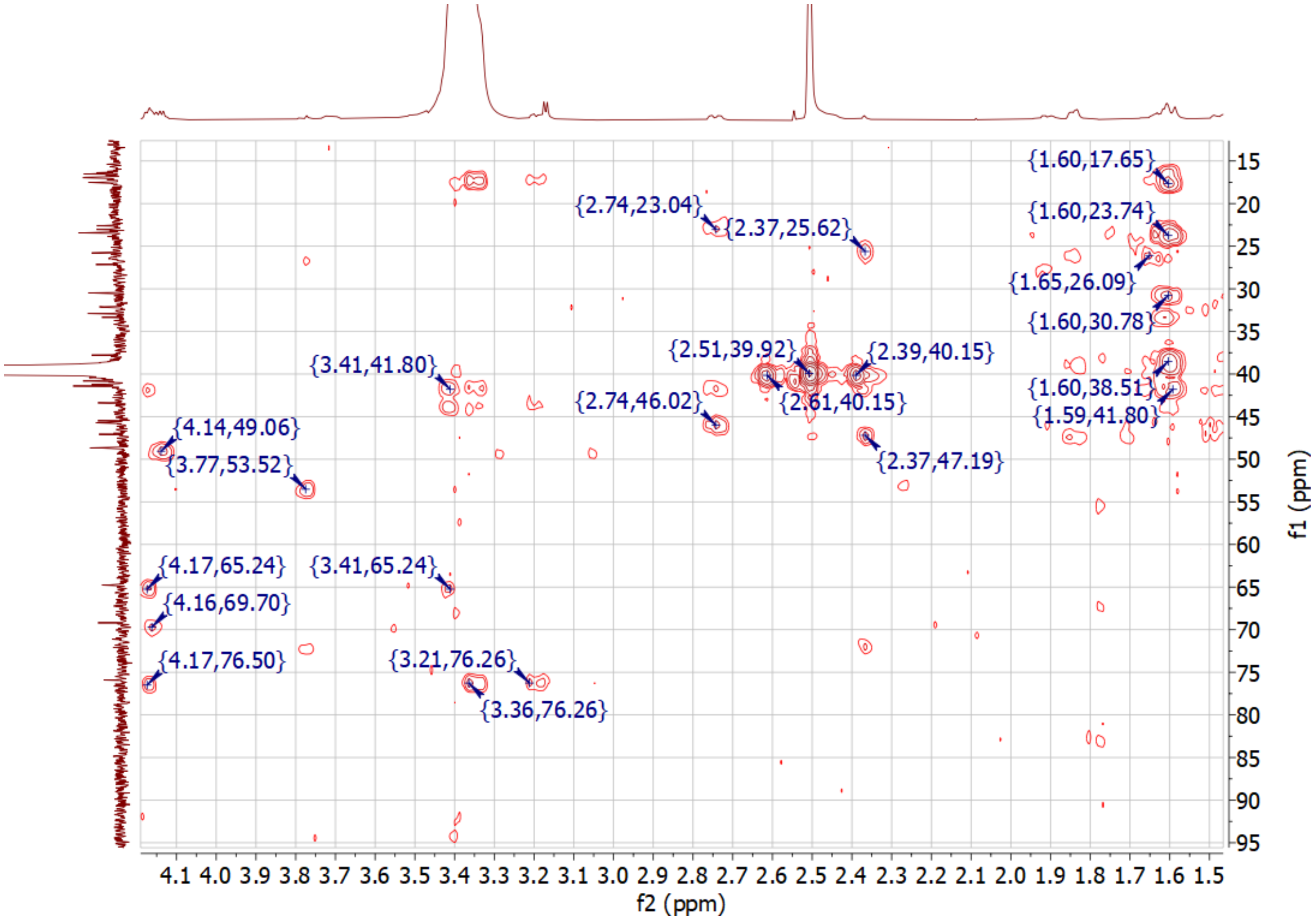

Figure S29. R_t 27.47 min peak ^{1}H-^{13}C NMR correlation (HMBC) spectrum, zoom over 15 to 95 ppm (f_1) and 1.50 to 4.20 ppm (f_2). Hederagenic acid.

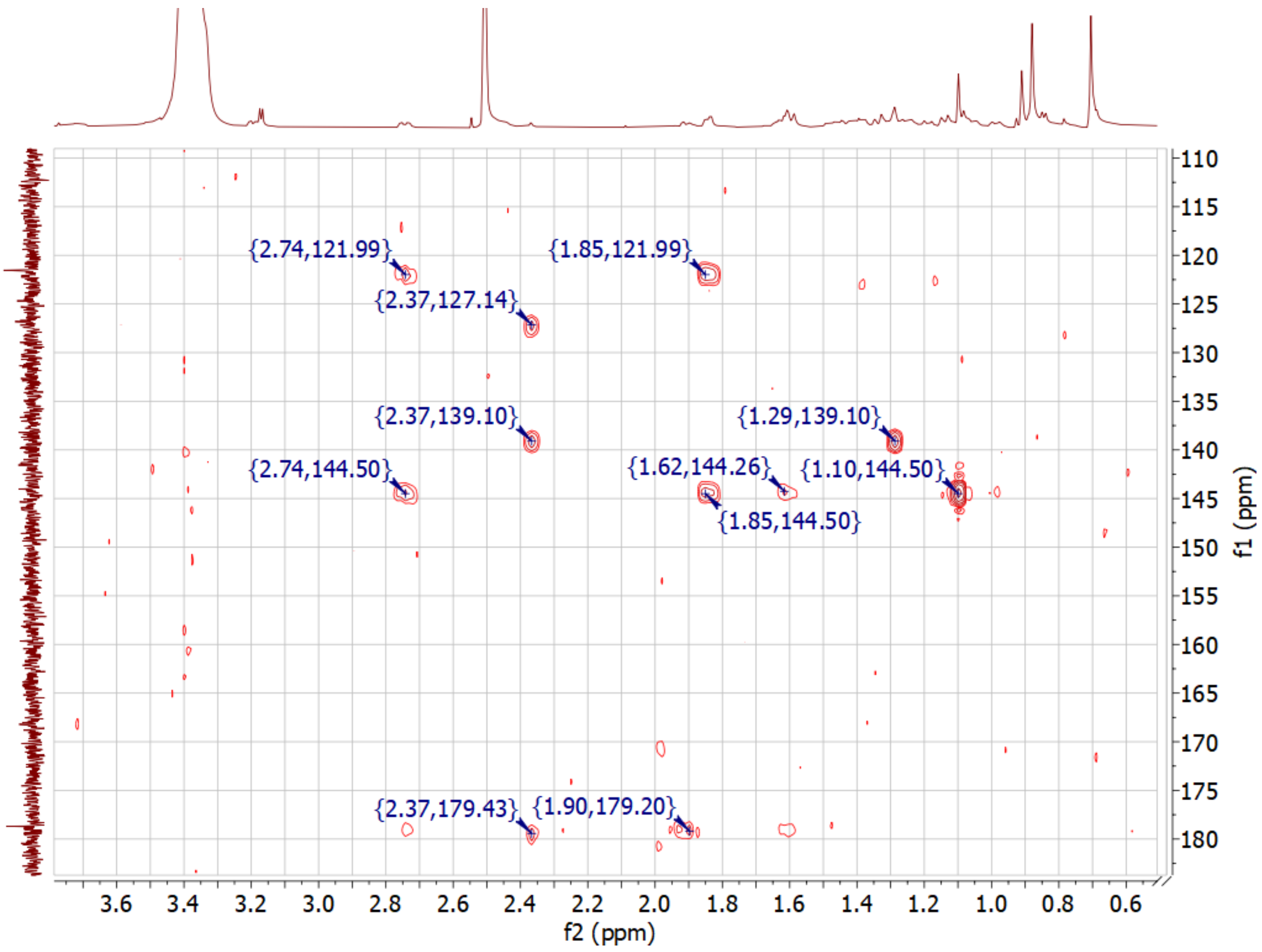

Figure S30. R_t 27.47 min peak ^{1}H-^{13}C NMR correlation (HMBC) spectrum, zoom over 110 to 180 ppm (f_1) and 0.60 to 3.60 ppm (f_2). Hederagenic acid.

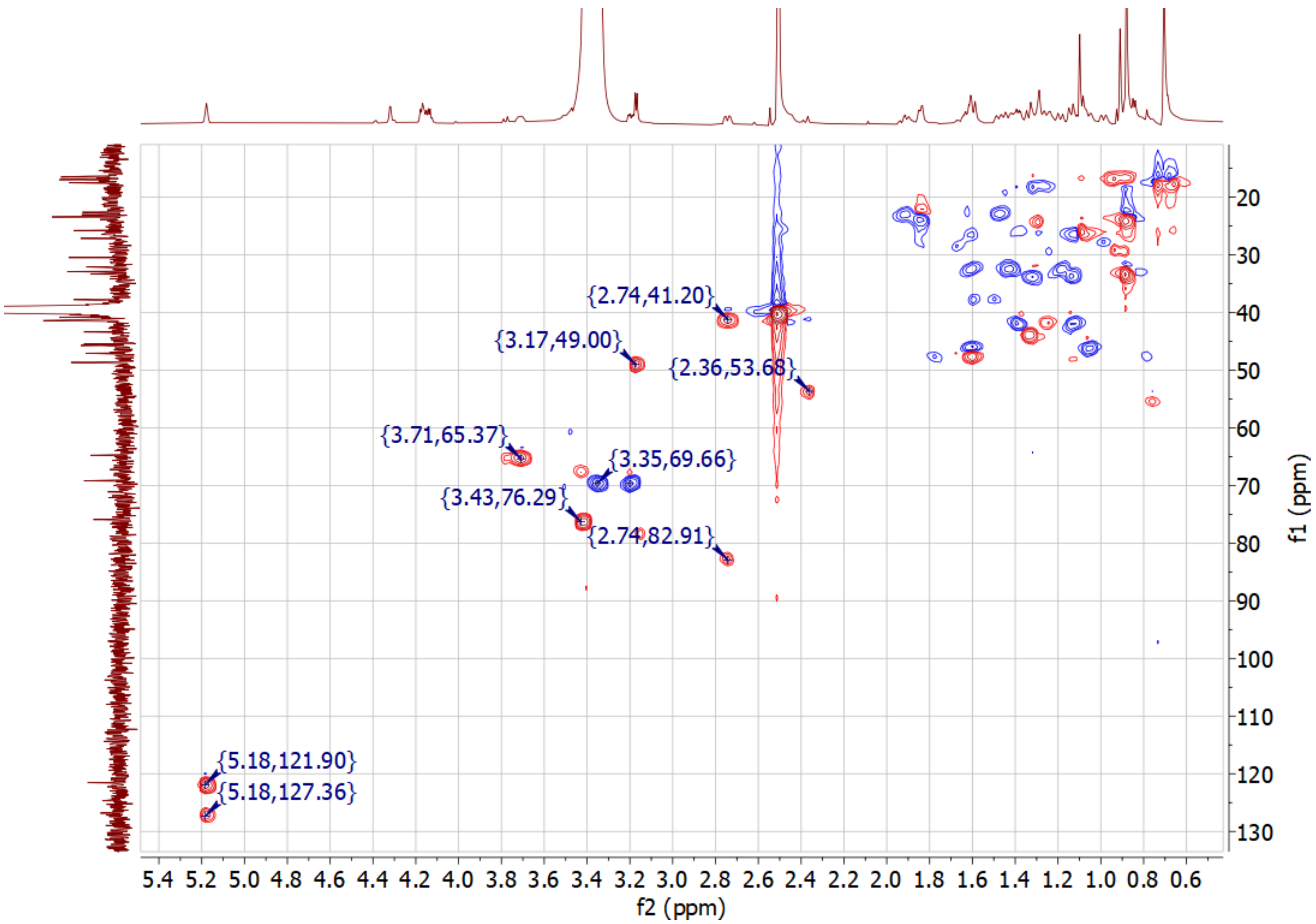

Figure S31. R_t 27.47 min peak ^{1}H-^{13}C NMR correlation (HSQC) spectrum. Hederagenic acid.

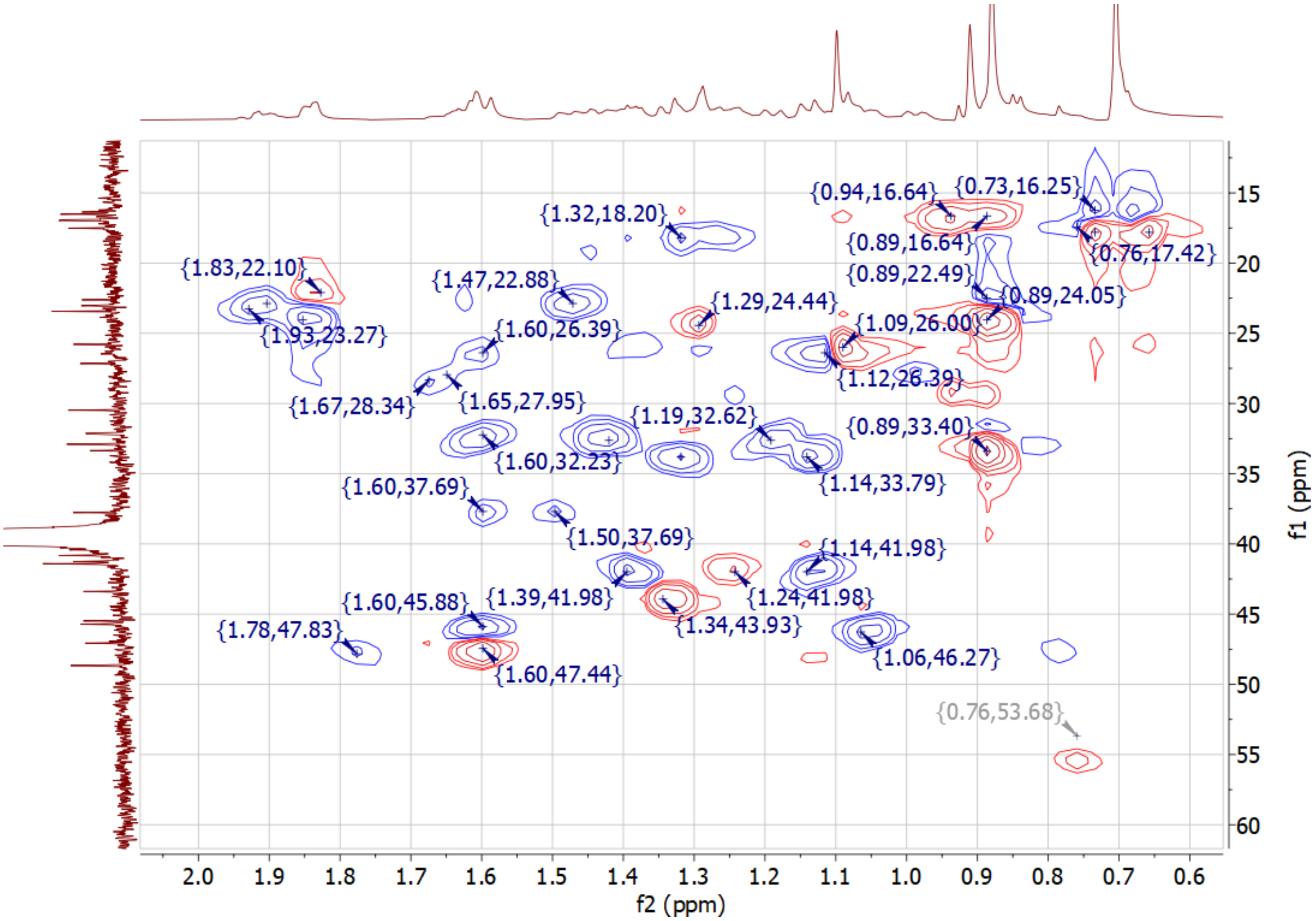

Figure S32. R_t 27.47 min peak ^{1}H-^{13}C NMR correlation (HSQC) spectrum, zoom over 15 to 60 ppm (f_1) and 0.60 to 3.0 ppm (f_2). Hederagenic acid.

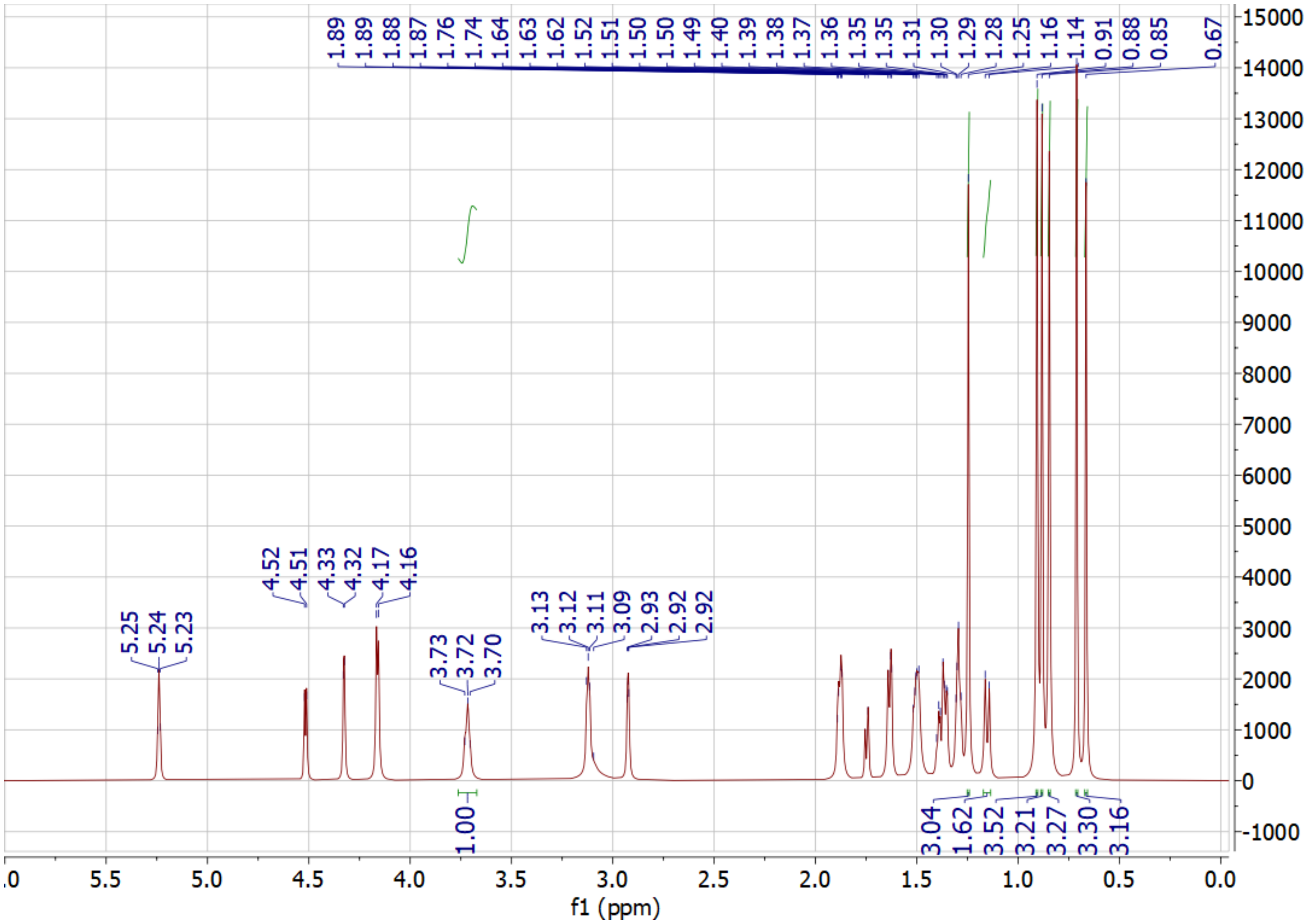

Figure S33. R_t 28.57 min peak ^{1}H-NMR spectrum. Arjunolic acid.

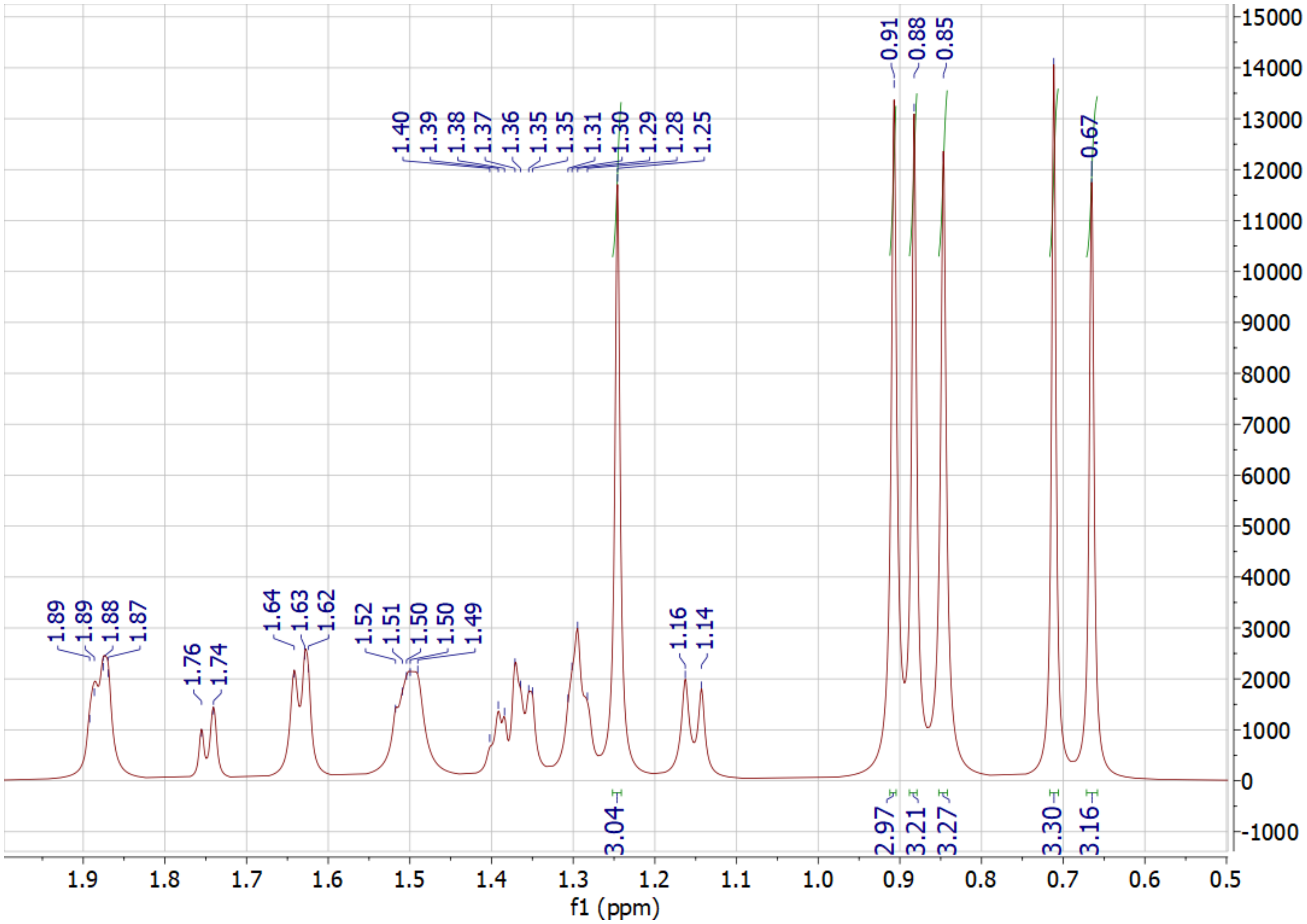

Figure S34. R_t 28.57 min peak ^{1}H-NMR spectrum, zoom over 0.5 to 1.9 ppm region. Arjunolic acid.

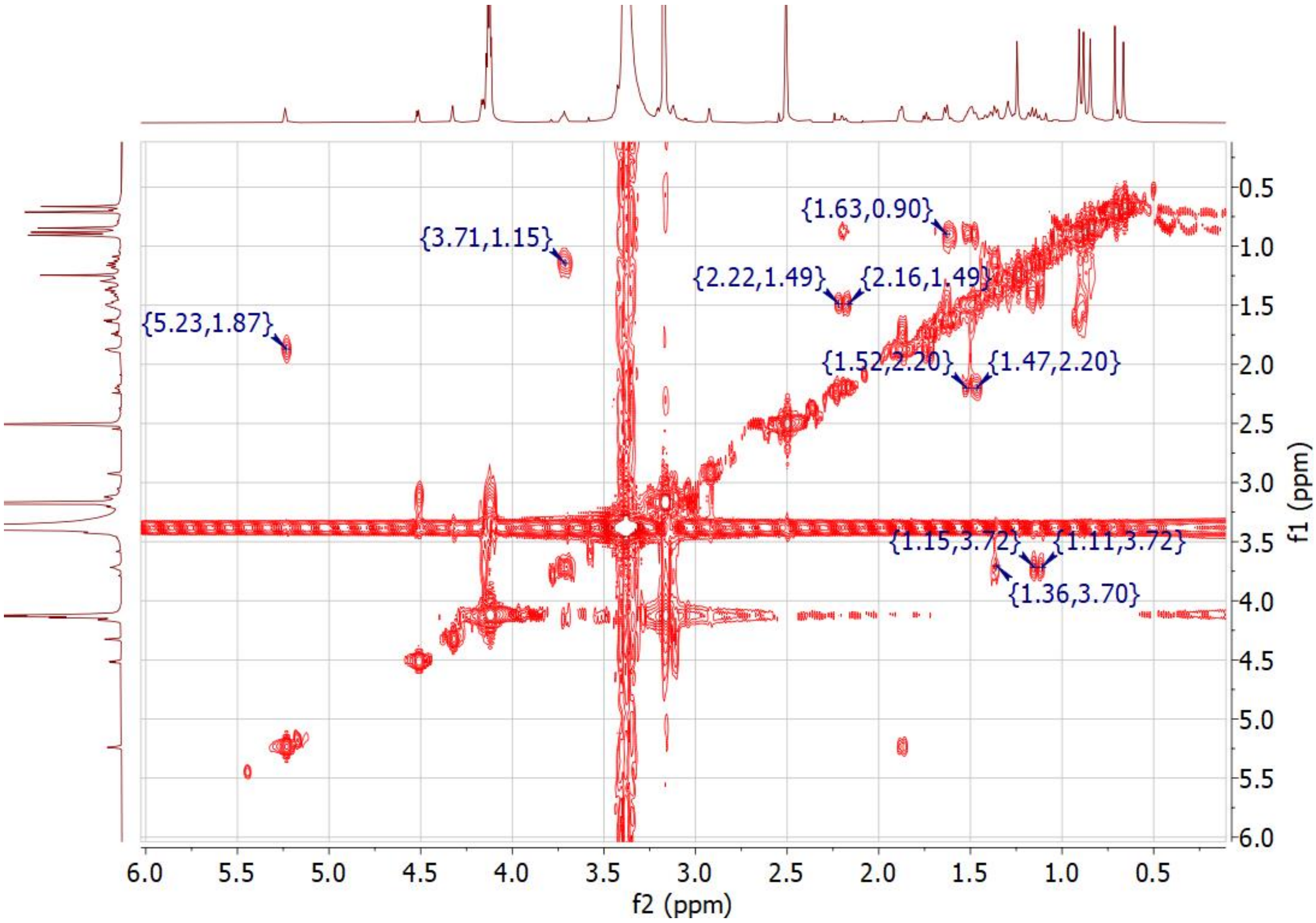

Figure S35. R_t 28.57 min peak peak ^{1}H-^{1}H NMR correlation (COSY) spectrum. Arjunolic acid.

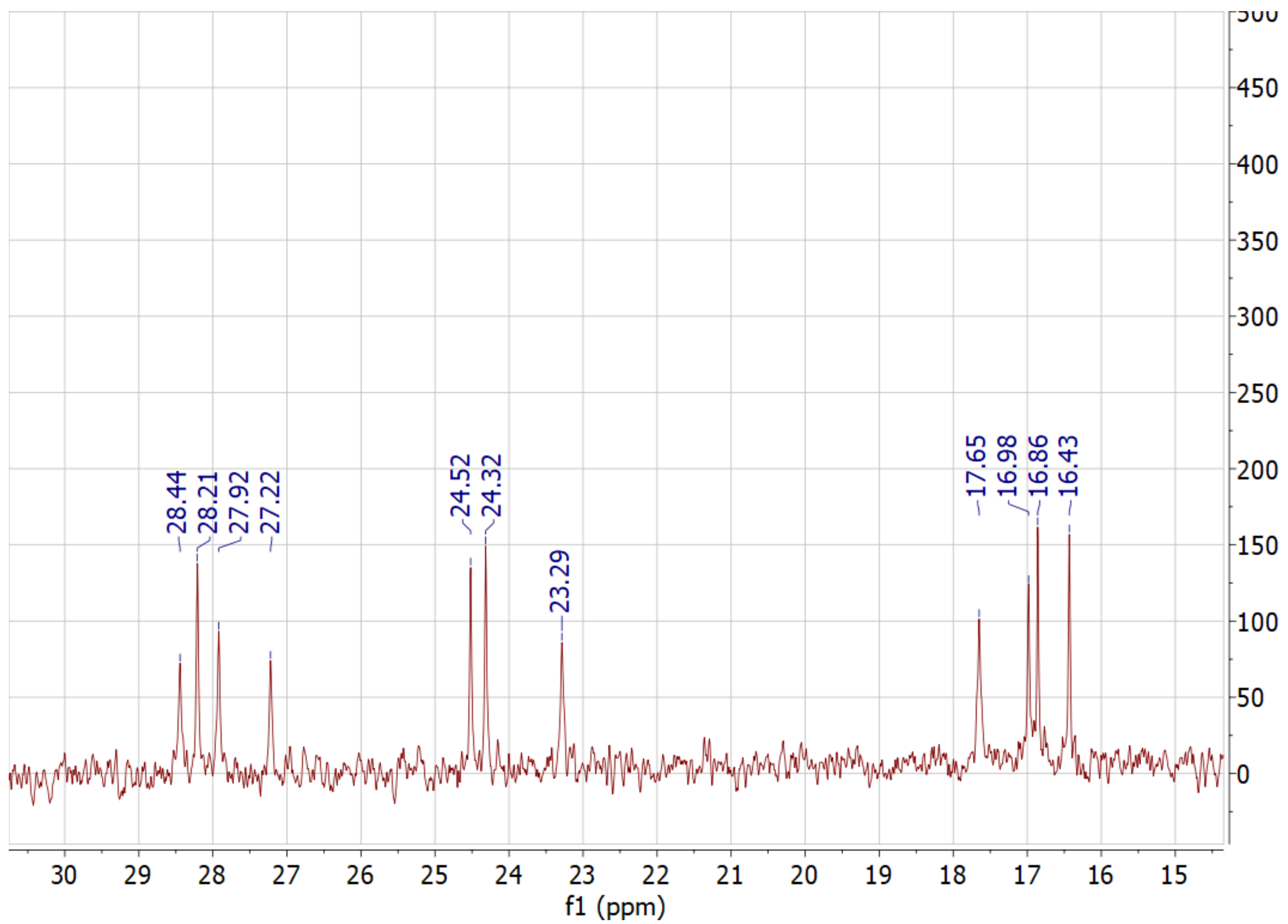

Figure S36. R_t 28.57 min peak peak ^{13}C-NMR spectrum, zoom over 15 to 30 ppm region. Arjunolic acid

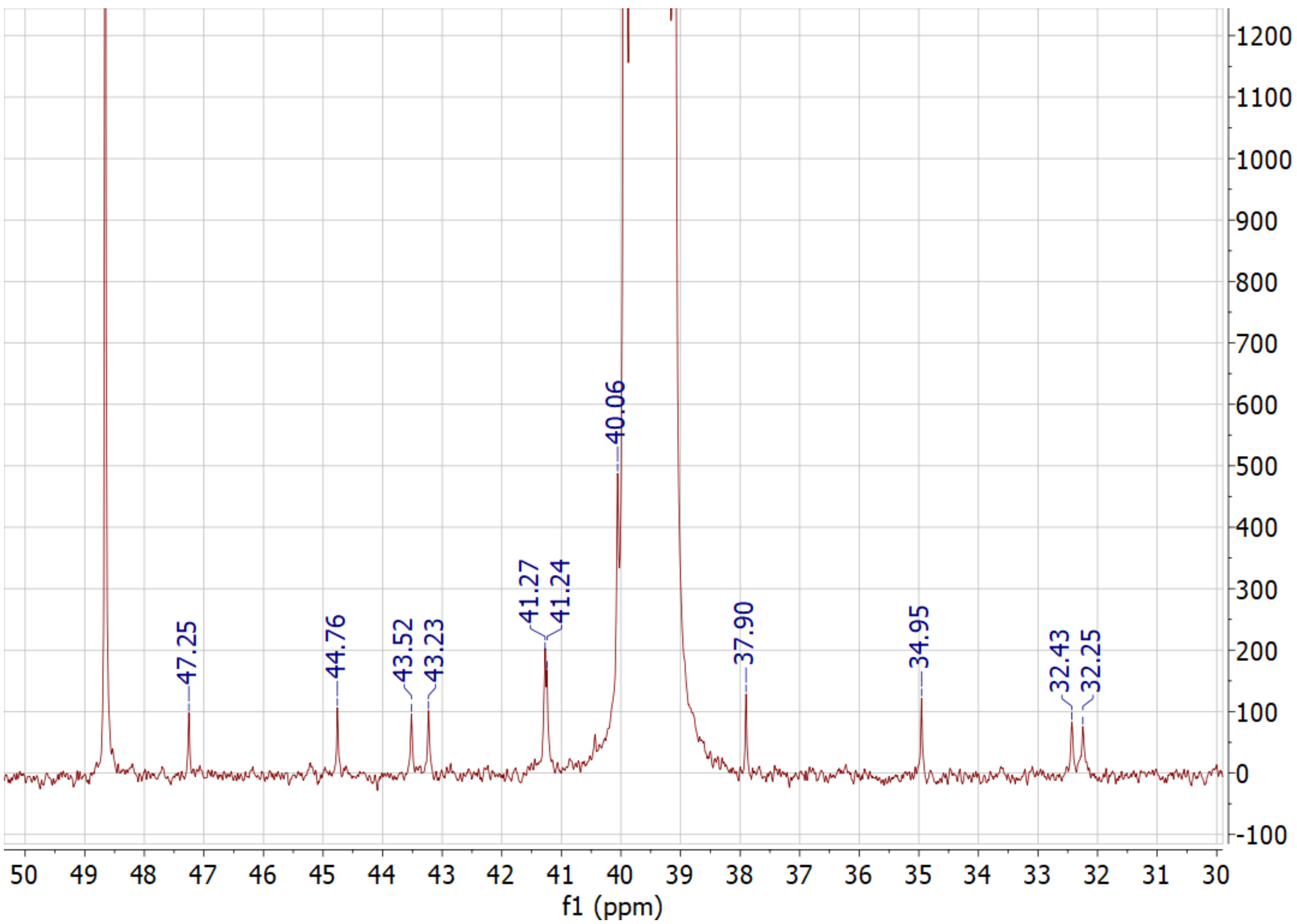

Figure S37. R_t 28.57 min peak peak ^{13}C-NMR spectrum, zoom over 30 to 50 ppm region. Arjunolic acid

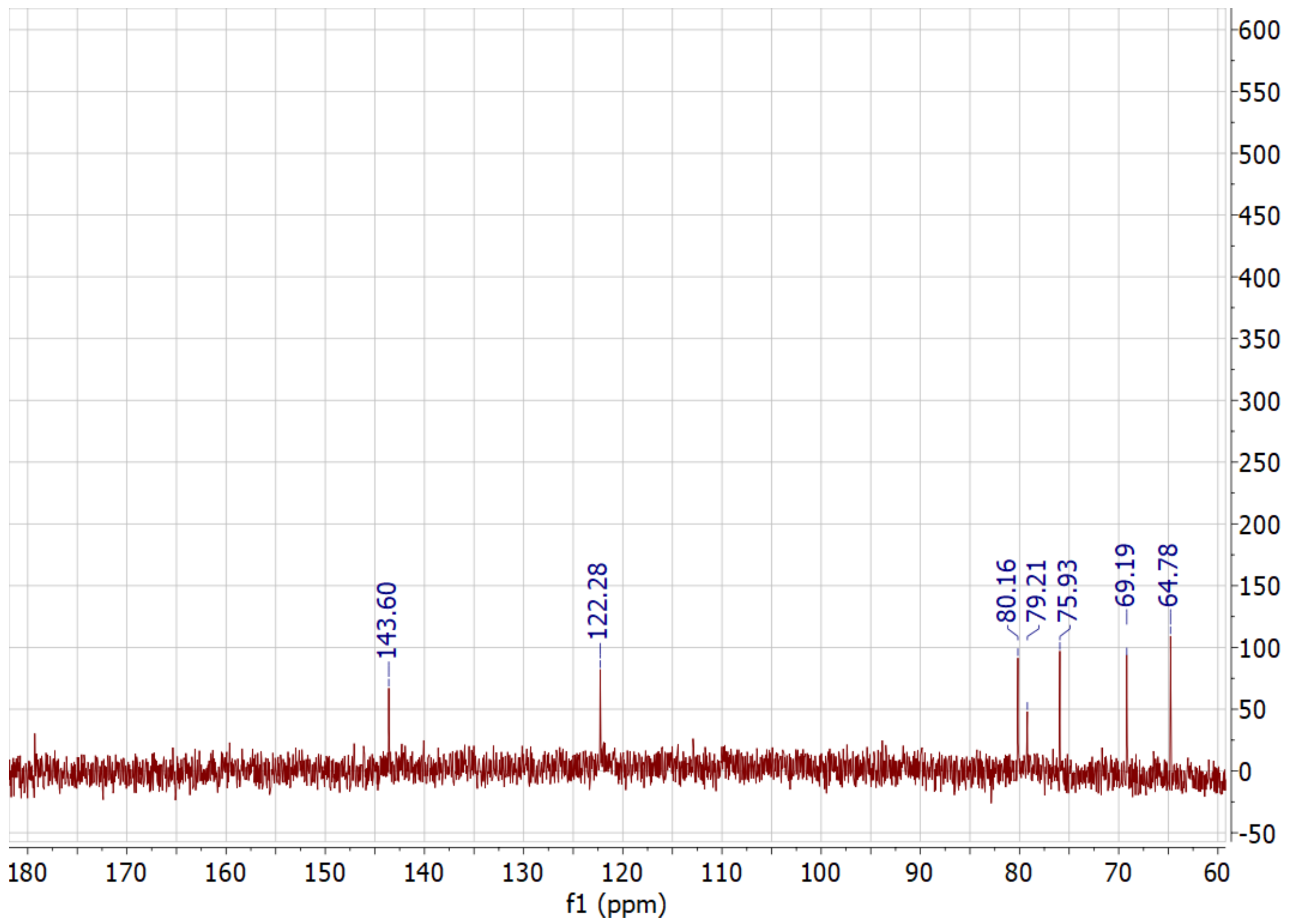

Figure S38. R_t 28.57 min peak peak ^{13}C-NMR spectrum, zoom over 60 to 180 ppm region. Arjunolic acid

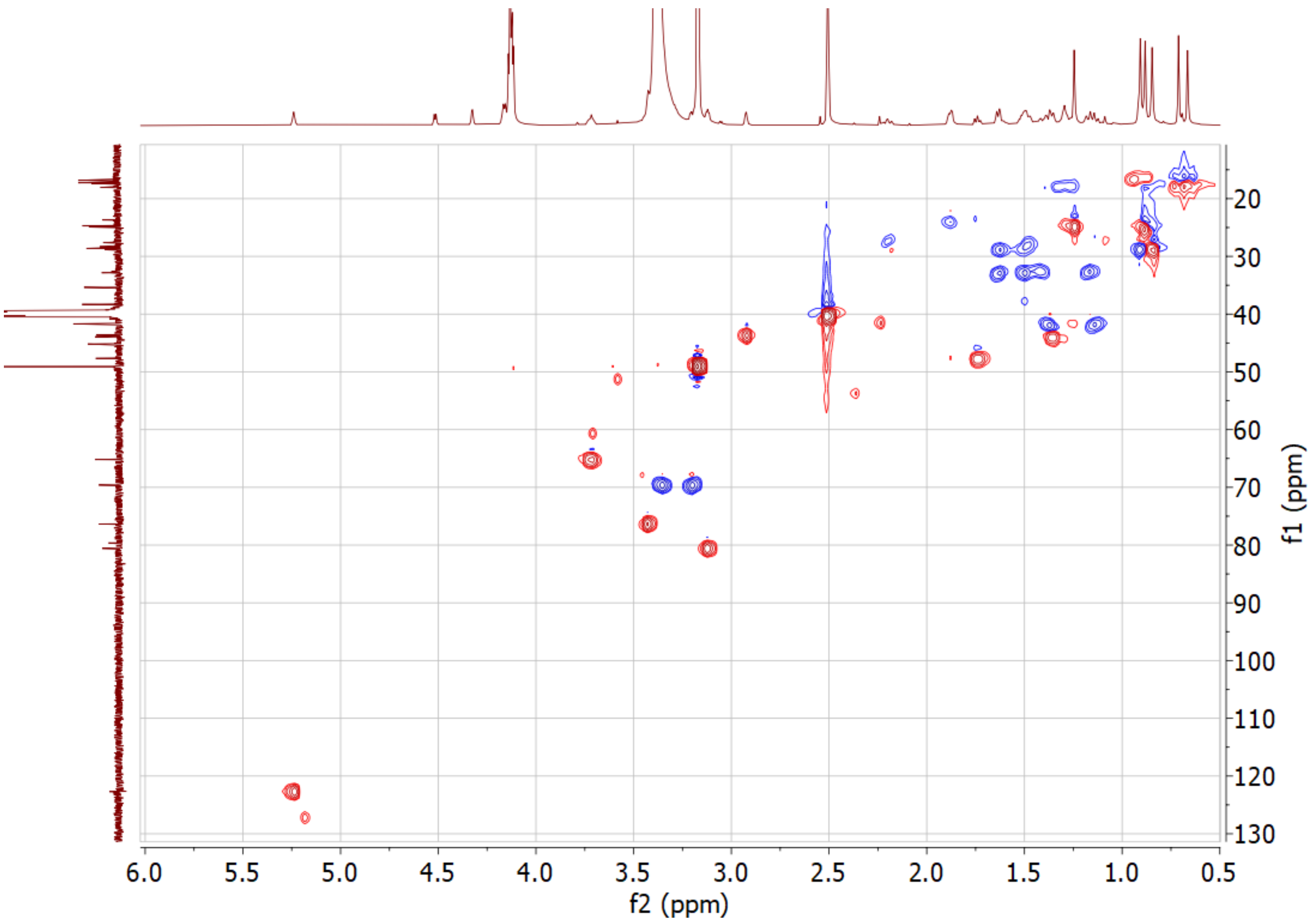

Figure S39. R_t 28.57 min peak peak ^{1}H-^{13}C NMR correlation (HSQC) spectrum. Arjunolic acid

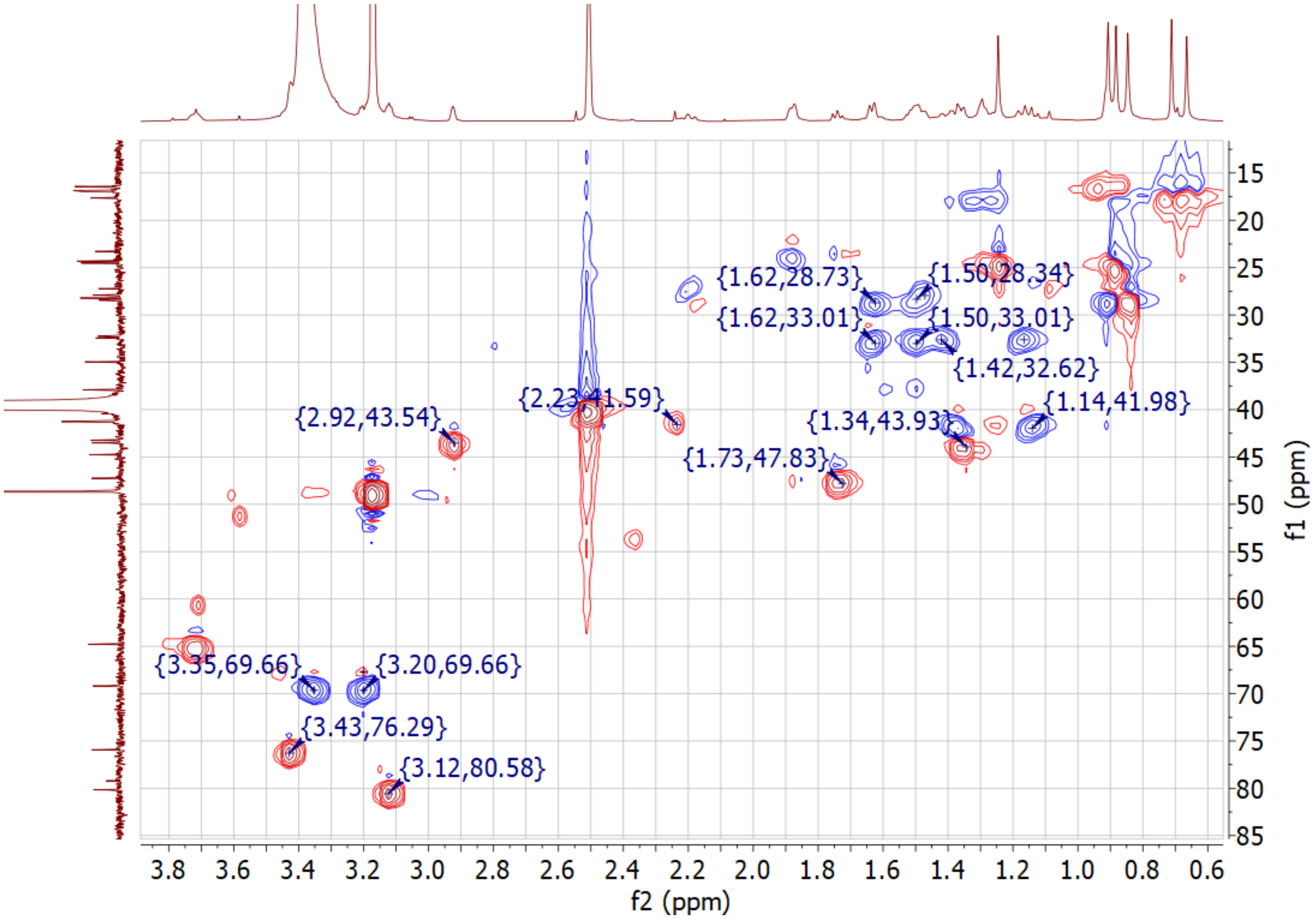

Figure S40. R_t 28.57 min peak peak ^{1}H-^{13}C NMR correlation (HSQC) spectrum, 15 to 85 ppm (f_1) and 0.60 to 3.8 ppm (f_2). Arjunolic acid

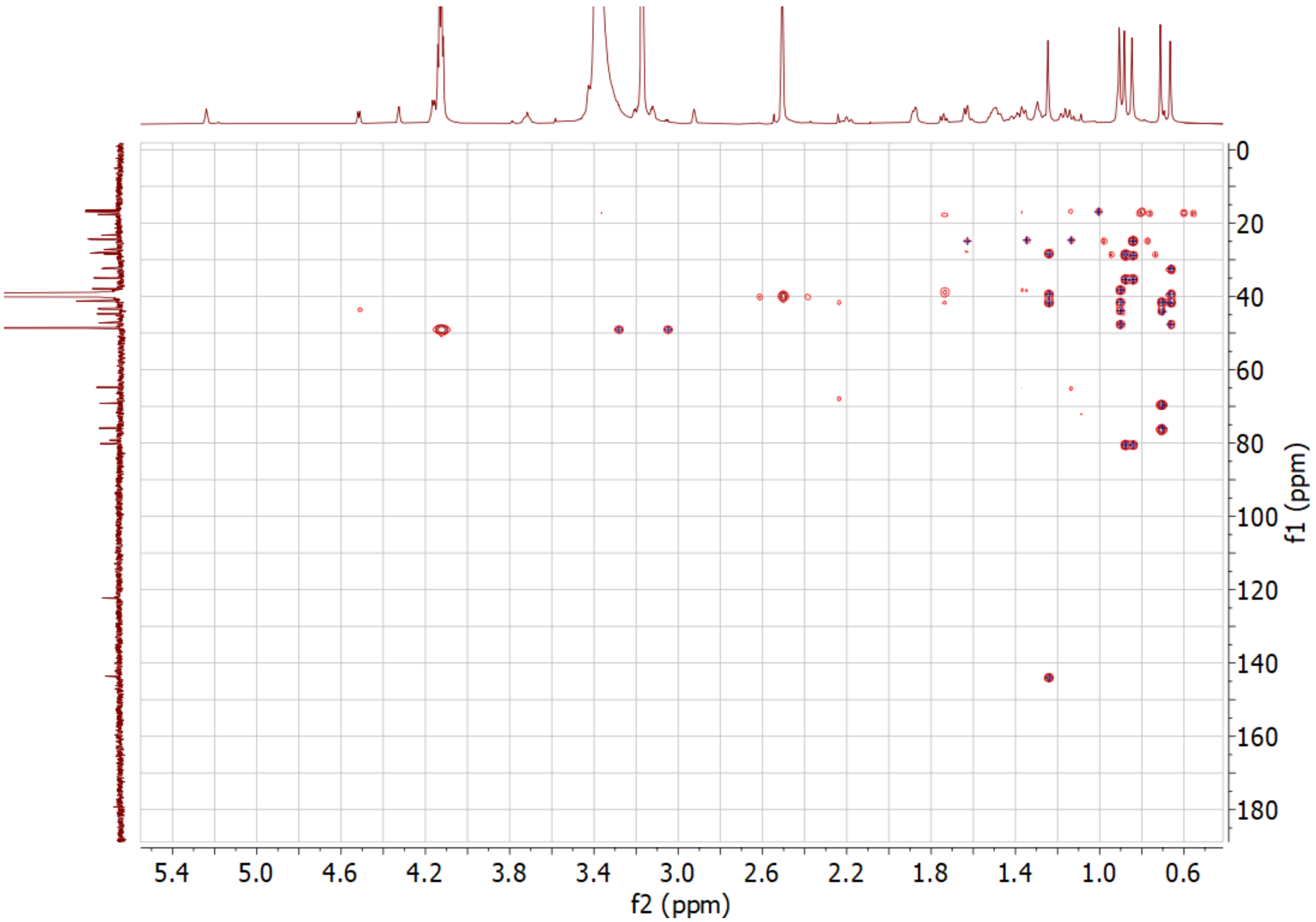

Figure S41. R_t 28.57 min peak peak ^{1}H-^{13}C NMR correlation (HMBC) spectrum. Arjunolic acid

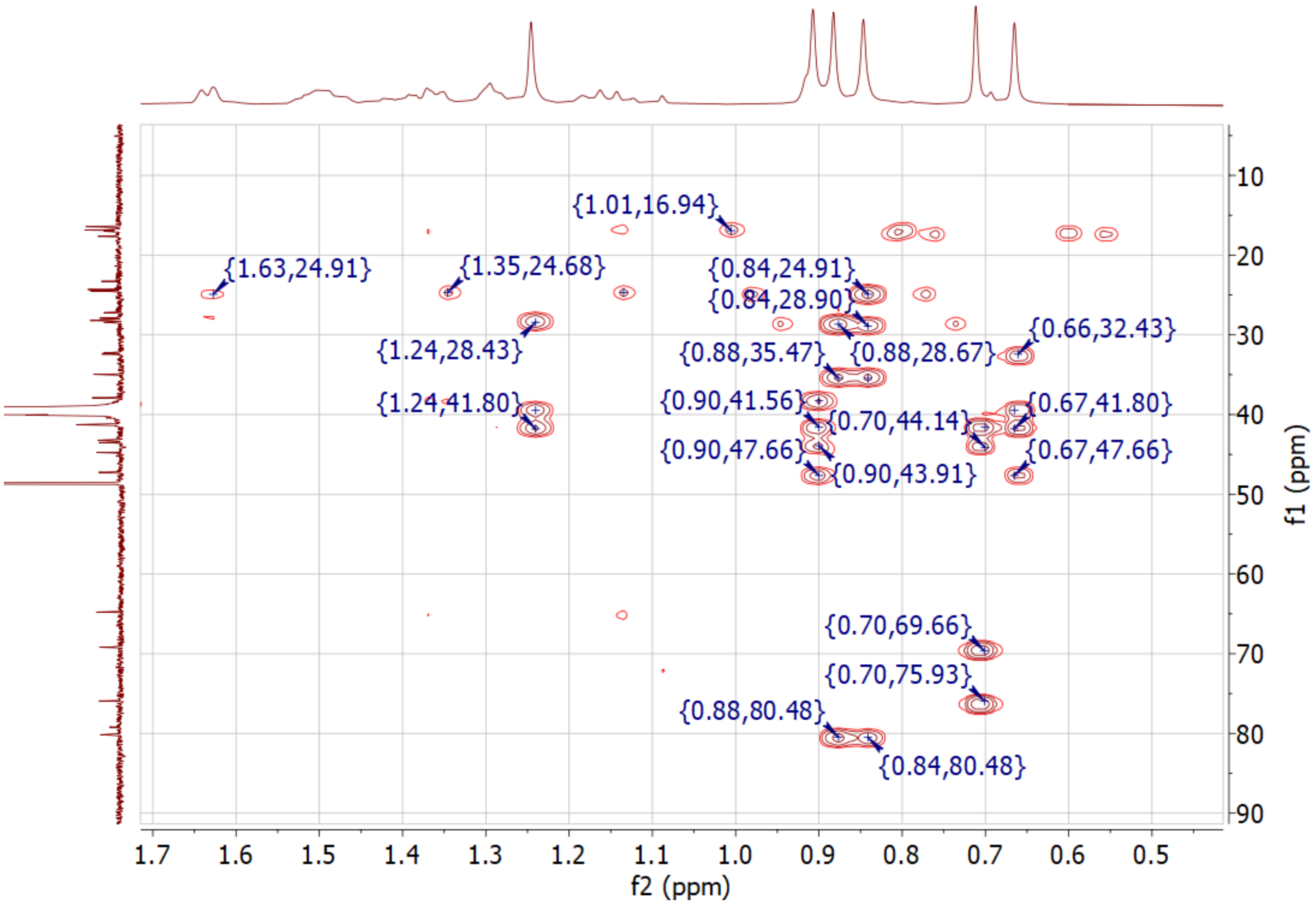

Figure S42. R_t 28.57 min peak peak ^{1}H-^{13}C NMR correlation (HMBC) spectrum, 10 to 90 ppm (f_1) and 0.50 to 1.7 ppm (f_2). Arjunolic acid
